# The roles of predictors in cardiovascular risk models - a question of modeling culture?

**DOI:** 10.1186/s12874-021-01487-4

**Published:** 2021-12-18

**Authors:** Christine Wallisch, Asan Agibetov, Daniela Dunkler, Maria Haller, Matthias Samwald, Georg Dorffner, Georg Heinze

**Affiliations:** 1 Section for Clinical Biometrics, Center for Medical Statistics, Informatics and Intelligent Systems, Medical University of Vienna, Spitalgasse 23, 1090 Vienna, Austria; 2 Section for Artificial Intelligence and Decision Support, Center for Medical Statistics, Informatics and Intelligent Systems, Medical University of Vienna, Vienna, Austria; 3 Department of Nephrology, Ordensklinikum Linz, Hospital Elisabethinen, Linz, Austria

**Keywords:** Cardiovascular risk, Prediction model, Predictors, Non-linear effect, Partial dependence plots

## Abstract

**Background:**

While machine learning (ML) algorithms may predict cardiovascular outcomes more accurately than statistical models, their result is usually not representable by a transparent formula. Hence, it is often unclear how specific values of predictors lead to the predictions. We aimed to demonstrate with graphical tools how predictor-risk relations in cardiovascular risk prediction models fitted by ML algorithms and by statistical approaches may differ, and how sample size affects the stability of the estimated relations.

**Methods:**

We reanalyzed data from a large registry of 1.5 million participants in a national health screening program. Three data analysts developed analytical strategies to predict cardiovascular events within 1 year from health screening. This was done for the full data set and with gradually reduced sample sizes, and each data analyst followed their favorite modeling approach. Predictor-risk relations were visualized by partial dependence and individual conditional expectation plots.

**Results:**

When comparing the modeling algorithms, we found some similarities between these visualizations but also occasional divergence. The smaller the sample size, the more the predictor-risk relation depended on the modeling algorithm used, and also sampling variability played an increased role. Predictive performance was similar if the models were derived on the full data set, whereas smaller sample sizes favored simpler models.

**Conclusion:**

Predictor-risk relations from ML models may differ from those obtained by statistical models, even with large sample sizes. Hence, predictors may assume different roles in risk prediction models. As long as sample size is sufficient, predictive accuracy is not largely affected by the choice of algorithm.

**Supplementary Information:**

The online version contains supplementary material available at 10.1186/s12874-021-01487-4.

## Background

Using cardiovascular disease (CVD) risk calculators is nowadays a daily routine in clinical practice when assessing a patient’s CVD risk profile. Widely used CVD risk prediction models such as the Framingham 2008 CVD risk model were statistically estimated by fitting a Cox model with a relatively small number of coefficients [1] and it is fully transparent how predictors impact on predictions. Although coefficients in risk prediction models have no causal interpretation, understanding how and why predictions differ between persons is an essential prerequisite for their widespread use. Statistical models may be extended to accommodate more complex predictor-risk associations if needed, which introduces flexibility to a strictly formula-based model by adding non-linear functions for continuous predictors or by adding interaction terms [2–4].

In machine learning (ML), explicit decisions on the model structure are intentionally avoided as it is believed that an algorithm ‘learns’ about the necessary complexity of the prediction model. Neural networks and extreme gradient boosting are two representatives of this way of ‘algorithmic’ modeling [5, 6]. An important caveat of many ML algorithms is that the final model structure is non-transparent and predictions seem to be generated by a ‘black-box’. This impedes reproducibility as well as quantification of a particular predictor-risk relation. Recently, the development of techniques to increase the transparency of ML models has become a highly active field of research [7, 8]. Several techniques have been proposed [9, 10], and some of them have been denoted as ‘model-agnostic’ as they can be applied without knowing how a modeling algorithm arrives at predictions.

Standard statistical modeling and ML use distinct approaches to generate predictions and the phrase ‘modeling culture’ was coined to describe these two fundamentally different paradigms of data analysis [11]. While several studies found similar predictive accuracy of prediction models developed under the paradigms of ML and standard statistical modeling [12, 13], none has shown how the choice of modeling paradigm may affect the interpretation of a predictor in a model at different data availabilities. Therefore, we aimed to visualize and compare the predictor-risk relation obtained by ML algorithms and standard statistical models in cardiovascular risk prediction. Our second aim was to demonstrate the impact of sample size on the shape and stability of the estimated relations.

## Methods

This study is reported according to the TRIPOD guidelines for model development and the checklist is provided as Additional file 1 [14].

### Study design

To exemplify the assessment of predictor-risk relations in practice, we reanalyzed a large registry study previously used to validate and update existing cardiovascular risk prediction models [15, 16] which represented our maximum (full) data availability. We simulated poorer data availabilities by gradually reducing the sample size by random subsampling.

Three authors were given information on the available predictors, outcomes and the expected event rate and received development datasets of various sample sizes. These three data analysts represented different modeling cultures according to their personal experience and training and developed models following their favorite ‘modeling paradigm’. In particular, CW (representing statistical modeling with generalized additive models), GD (ML with neural networks), and AA (ML with boosting) had to accomplish the following two tasks independently:

1. To develop an analysis strategy following their favorite paradigm. Depending on sample size amendments to the analysis strategy were allowed.
2. To develop prediction models and to provide a prediction tool for each of the models to facilitate individual calculation of predictions as usually required for bedside use.

Using the provided prediction tools, GH and DD assessed the resulting predictor-risk relation and evaluated predictive accuracy in an independent test set.

### Study population

Our pseudonymized database comprised electronic health records from the Austrian preventive health screening program (1/2009–3/2014) including measurements on the predictors included in the Framingham 2008 CVD risk model and other known or assumed CVD risk predictors. These data were linked to data on hospitalizations (1/2008–3/2015) and causes of death (1/2009–3/2015) from the same individuals to determine if a CVD event had occurred after the first health screening. Data preparation steps have been reported previously [15, 16], and relevant additions to the current work are detailed in Additional file 2: Appendix 1. We applied the inclusion criteria of the Framingham 2008 CVD risk model, where individuals between 30 and 74 years who had no indication of CVD in the year prior to the health screening were included [1, 15, 16]. Moreover, we required participants to have at least 1 year of follow-up. The study protocol and the exempt from the need to obtain informed consent was approved by the Ethics Committee of the Medical University of Vienna (ECS 1232/2014).

### Outcome

We used the occurrence of any cardiovascular event within 1 year after the health screening as our outcome variable. Cardiovascular events were defined in line with the Framingham 2008 CVD risk model as the diagnosis of any of: coronary heart disease (coronary death, myocardial infarction, coronary insufficiency, angina), cerebrovascular events (ischemic stroke, hemorrhagic stroke, transient ischemic attack), peripheral artery disease (intermittent claudication), or heart failure [1]. Appropriate ICD-10 codes (10th revision of the International Classification of Diseases) for CVD were used to identify cases [15, 16]. Further information on the identification of CVD in our study cohort is detailed in Additional file 2: Appendix 1.

### Predictors

Similar to the Framingham 2008 CVD risk model, we considered the following predictors: sex, age, total cholesterol (mg/dl), HDL cholesterol (mg/dl), systolic blood pressure (BP, mmHg), hypertensive drug intake (yes; no), diabetes (yes; no), and smoking status (yes; no). Moreover, the electronic health records also contained several other variables which we considered potentially relevant for cardiovascular risk prediction according to domain experts. These were: blood glucose (mg/dl), triglycerides (mg/dl), diastolic BP (mmHg), BMI score (kg/m^2^), glucose in urine (positive; negative), protein in urine (positive; negative), waist circumference (categorical; too large: waist circumference ≥ 102 cm for men or ≥ 88 cm for women; normal: < 102 cm for men or < 88 cm for women), self-assessed physical activity (none; occasionally; regularly), ratio of total cholesterol and HDL cholesterol, BP classes (categorical; ideal: < 120/80; normal: 120–129/80–85; still normal: 130–139/85–89; hypertension stage 1: 140–179/90–109; hypertension stage 2: ≥180/110; isolated systolic hypertension: ≥140/< 90), and BMI classes (categorical; < 18.5; 18.5–24.9; 25.0–29.9; 30.0–34.9; 35.0–39.9; ≥40.0).

**Fig. 1:**
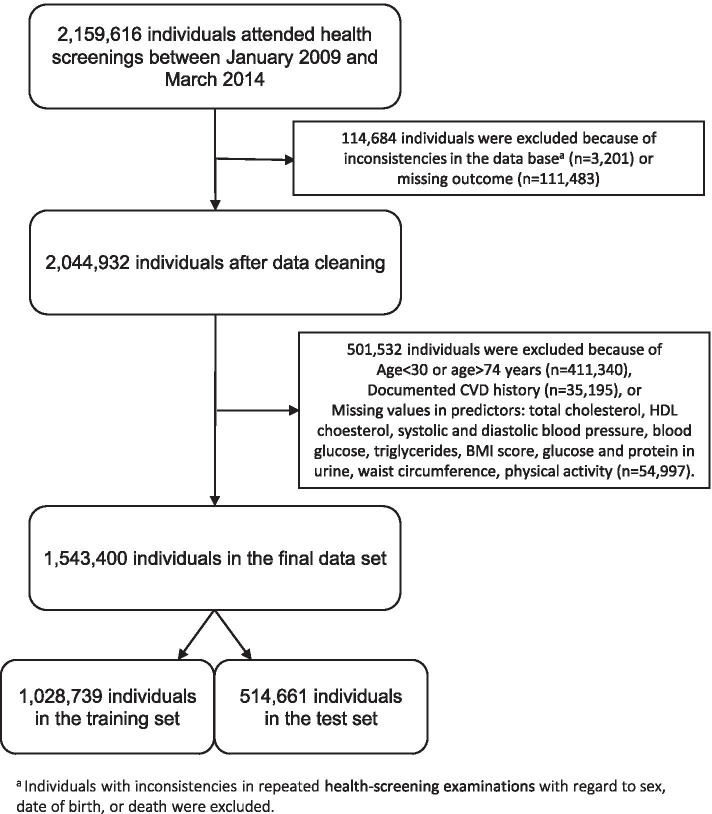
Flow chart of selection of individuals for this study. Abbreviations: BMI, body mass index; CVD, cardiovascular disease; HDL, high density lipoprotein

Starting with 2,159,616 individuals in the data base, we applied the inclusion criteria of Framingham 2008 CVD risk model and excluded individuals with missing values in any of the Framingham predictors or other potential predictors or outcome (Fig. 1). The resulting data set comprised 1,543,400 individuals with 17 predictors, and was randomly split into a training set (1,028,739 individuals) and a test set (514,661 individuals).

### Data availability: varying the sample size

To mimic analysis scenarios with various sample sizes available for model development, we defined five levels of data availability as follows: the full dataset (N; *n* = 1,028,739), half of the dataset (N/2; n ≈ 513,370), one-tenth (N/10; n ≈ 102,874), one-twenty-fifth (N/25; n ≈ 41,150), and one-hundredth of the dataset (N/100; n ≈ 10,287). For data availabilities of N/2, N/10, N/25 and N/100, the training set was split randomly into the corresponding number of approximately equally sized disjoint subsets. Hence, this resulted in two subsets at a data availability of N/2, and accordingly in ten, 25, and 100 subsets at N/10, N/25 and N/100. The full dataset and each of the 137 subsets were treated just like they were separate studies and served as training sets for model development.

### Data analysis

#### Statistical modeling with generalized additive models (GAM)

Logistic regression models are used to relate a binary outcome like the occurrence of a CV event to a set of predictors [17]. Similarly to linear regression, predictor values are combined through their weighted sum resulting in a so-called ‘linear predictor’. The linear predictor is then transformed by the logistic function to produce a probability that is naturally bounded between 0 and 1. Generalized additive models (GAM) [18] transform continuous predictors via splines [2, 19] allowing for a smooth, flexible and non-linear representation of these predictors in the linear predictor [20]. To introduce even more flexibility, interaction (product) terms can be added to the model. Model building may also include stepwise variable selection to reduce the number of estimated coefficients in a model [3, 21].

#### ML with neural networks

Neural networks connect predictors to the outcome through a network [5]. The edges of this network are the so-called weights similar to coefficients in a logistic regression model. A so-called single-layer neural network consists of one layer, the ‘input layer’ of predictors, and is equivalent to a logistic regression model (SLNN-LR). The idea can be extended by introducing a second, ‘hidden’ layer with ‘hidden units’ between the input layer and the outcome (multi-layer neural network, MLNN). This introduces more flexibility and the possibility of complex interactions between predictors [5]. The number of hidden units can either be specified in advance or optimized using cross-validation.

#### ML with boosting

Boosting is a general concept in ML in which a sequence of weak models is estimated such that each additional element in the sequence improves on the inaccuracies of its predecessors [22]. With extreme gradient boosted trees (XGBoost), simple decision trees are used as the elements in that sequence [23]. The final prediction is the average of the predictions made by all trees. The number of trees, the number of branches in each tree and other characteristics of the trees are so-called hyperparameters of the model.

### Predictive performance as requirement for interpretability

#### Predictive performance

The predictive performance of the final prediction models was assessed in the test set by the Brier score measuring the accuracy of prediction [24], and by measures of discrimination and calibration. As age is the most important predictor of cardiovascular events [16], the Brier score was also computed for different ages. Discrimination was quantified by the discrimination slope [25], the area under the receiver operating characteristic curve (AUROC) and the area under the precision-recall curve (AUPRC). Calibration was assessed visually by grouping the predicted probabilities by their permilles (1000-quantiles) and plotting a loess smoother through the 1000 observed risks corresponding to these 1000 groups defined by the permilles.

#### Agreement of predictions

Spearman’s rank correlation coefficients between predictions in the test set obtained from the different analytical strategies were computed pairwisely. For data availabilities of N/2, N/10, N/25 and N/100, the correlation coefficients were averaged at each level of data availability.

### Evaluation criterion: assessing the predictor-risk relation

We chose partial dependence plots (PDPs) and individual conditional expectation (ICE) plots as model-agnostic techniques to illustrate the direct effect of each predictor on the predictions of a model [9, 10, 23]. ICE plots show how predictions of a model result from varying the values of one predictor, keeping all other predictors fixed. For example, to construct an ICE plot for cholesterol, we used the combination of values of all other predictors observed for a particular patient, varied cholesterol across its observed range, and connected the resulting risk predictions. This was repeated for all patients of a reference population. PDP plots were then computed by averaging the ICE predictions at each cholesterol value and connecting the averages. In some PDP plots we fixed some important predictors at predefined values, e.g., we set age to 40, 50, 60 and 70 years, and sex to ‘female’ or ‘male’.

To reduce the computational burden, we generated a reference population of 10,000 individuals by randomly selecting 1000 men and 1000 women from each of five age groups (defined as 30–38; 39–47; 48–56; 57–66; 65–74 years) of the full training set. We then assigned weights to each sex-age group according to the corresponding prevalence in the training set. We used these weights when averaging predictions for PDPs.

## Results

### Analytical strategies

Concerning standard statistical modeling with GAMs, restricted cubic splines were used to model continuous predictors [2]. We considered background knowledge from previous publications on the importance of particular predictors and on the plausibility of interactions between predictors [1, 15, 16]. For lower levels of data availability, we gradually reduced the complexity of the models to meet the rule of thumb that for each regression coefficient to be estimated, at least 10 events should be available in the training set. For example, we fitted separate models for men and women at full data availability, at N/2 and at N/10. At data availabilities of N/25 and N/100, sex was a binary predictor in a combined model. Based on recent recommendations from Riley et al. (2020) [26], the number of observations at each data availability was sufficient for our considerations of flexibility. The sample size calculations, general information on the analytical strategies, more details on predictors used at each data availability are given in Additional file 2: Appendix 2 and 3a. The risk equations estimated by GAMs based on full data availability for men and women are found in Additional file 3.

To adapt the analytical strategy using neural networks to data availability, the number of hidden units was specified such that for each weight in the network, at least 10 events were available. At N/25 and N/100 this could not be achieved and some predictors were omitted based on prior assumptions about their low prognostic value fueled by background knowledge (Additional file 2: Appendix 3b). Both SLNNs and MLNNs were considered as two different analytical strategies with different flexibilities.

For XGBoost, the analytical strategy was not altered depending on data availability. For each training set, 5-fold cross-validation was performed to find the optimal values for all hyperparameters based on AUROC. The optimal configuration of hyperparameters, which is found in Additional file 2: Appendix 3c, was then used to fit XGBoost on that training set.

### Patients

Characteristics of individuals in the training and test sets at baseline were almost identical (Table 1, Additional file 2: Appendix 4). There were slightly more women (53.8%) than men (46.2%), and women had more favorable baseline characteristics; e.g., they exhibited lower prevalence of smoking or diabetes. Women also had higher HDL cholesterol, lower triglyceride values, and lower systolic and diastolic blood pressure. The primary endpoint was observed in 9770 (0.9%) and 4802 (0.9%) individuals in the full training set and in the test sets, respectively.

**Table 1:** Baseline characteristics of individuals in the test set. Continuous variables are reported as mean (sd) and for categorical variables absolute numbers and percentages are given

	Test set (***n*** = 514,661)	
	Men (***n*** = 237,748; 46.2%)	Women (***n*** = 276,913; 53.8%)
**Age (years)**	49.9 (11.9)	50.1 (12.2)
**Total cholesterol (mg/dl)**	210 (41.9)	212 (41.6)
**HDL cholesterol (mg/dl)**	51.4 (14.9)	63.9 (17.2)
**Cholesterol ratio (total cholesterol/HDL cholesterol)**	4.38 (1.43)	3.54 (1.14)
**Triglycerides (mg/dl)**	146 (94.6)	108 (62.3)
**Blood glucose (mg/dl)**	98.3 (24.7)	92.7 (20.4)
**Systolic BP (mmHg)**	133 (17.3)	127 (18.7)
**Diastolic BP (mmHg)**	82.7 (10.1)	79.6 (10.4)
**BP classes**		
Ideal	25,964 (11.0%)	67,687 (24.4%)
Normal	59,743 (25.1%)	74,538 (26.9%)
Still normal	55,766 (23.5%)	52,224 (18.9%)
Hypertension stage 1	56,417 (23.7%)	45,370 (16.4%)
Hypertension stage 2	7200 (3.0%)	6264 (2.3%)
Isolated systolic hypertension	32,658 (13.7%)	30,830 (11.1%)
**Hypertensive drug intake**		
Yes (vs. no)	32,731 (13.8%)	34,987 (12.6%)
**Smoking status**		
Yes (vs. no)	59,279 (24.9%)	58,164 (21.0%)
**Diabetes**		
Yes (vs. no)	13,688 (5.8%)	11,006 (4.0%)
**BMI score (kg/m**^**2**^**)**	27.4 (4.69)	26.0 (5.68)
**BMI classes**		
< 18.5	866 (0.4%)	6546 (2.4%)
18.5–24.9	75,942 (31.9%)	136,837 (49.4%)
25.0–29.9	108,615 (45.7%)	78,301 (28.3%)
30.0–34.9	37,675 (15.8%)	34,310 (12.4%)
35.0–39.9	8229 (3.5%)	11,454 (4.1%)
≥ 40.0	6421 (2.7%)	9465 (3.4%)
**Waist circumference**		
Too large (vs. okay)	83,503 (35.1%)	96,416 (34.8%)
**Physical activity**		
None	26,347 (11.1%)	31,161 (11.3%)
Ocassionally	101,706 (42.8%)	118,890 (42.9%)
Regularly	109,695 (46.1%)	126,862 (45.8%)
**Protein in urine**		
Positive (vs. negative)	14,403 (6.1%)	16,267 (5.9%)
**Glucose in urine**		
Positive (vs. negative)	5481 (2.3%)	4294 (1.6%)

*Abbreviations*: *BMI* body mass index, *BP* blood pressure, *HDL* high density lipoprotein

### Predictive performance

**Fig. 2:**
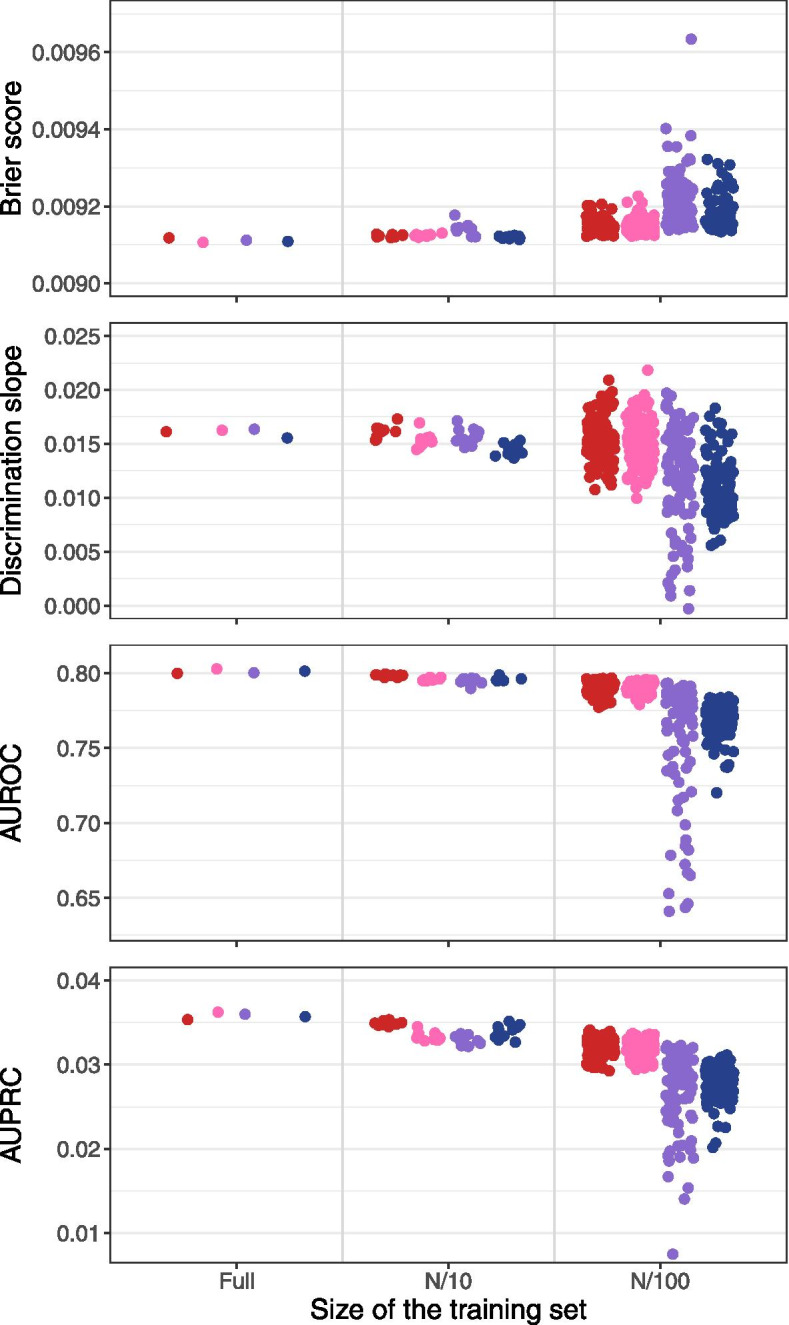
Brier Score, discrimination slope, AUROC and AUPRC of the models (SLLN-LR (red), GAM (rose), MMLN (violet), XGBoost (blue)) fitted at full data availability, data availability of N/10 and N/100, evaluated in the test set. Abbreviations: GAM, generalized additive models; MLNN, multi-layer neural networks; SLNN-LR, single-layer neural network/logistic regression; XGBoost, extreme gradient boosted trees

**Fig. 3:**
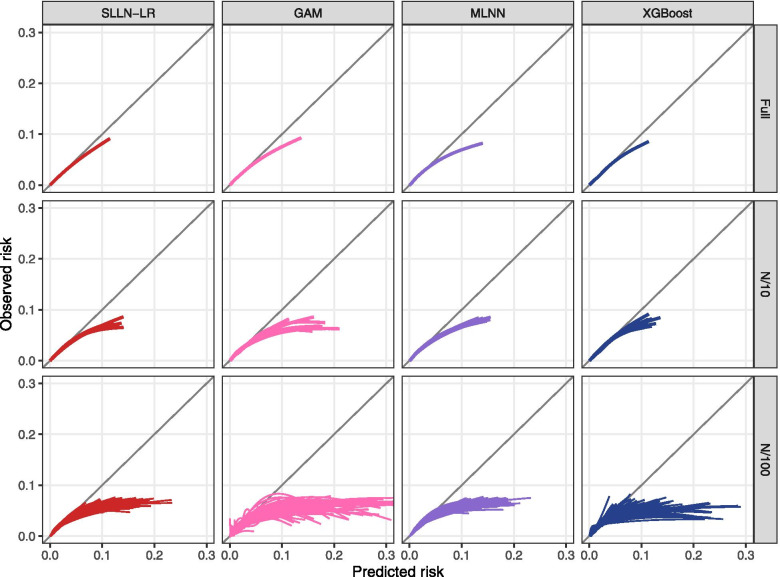
Calibration of the models (SLLN-LR, GAM, MLNN, XGBoost) fitted at full data availability, data availability of N/10 and N/100, evaluated in the test set. The visualization across the full range of predicted risk is found in Additional file 2: Appendix 6. Abbreviations: GAM, generalized additive models; MLNN, multi-layer neural networks; SLNN-LR, single-layer neural network/logistic regression; XGBoost, extreme gradient boosted trees

At full data availability, all analytical strategies yielded approximately equal predictive accuracies on the test set with respect to AUROC (range 0.8000 to 0.8029), AUPRC (range 0.03535 to 0.03624), Brier scores (0.0091 for all analytical strategies) and discrimination slopes (0.0156 to 0.0164) (Fig. 2). These numbers indicate discrimination similar to widely used prediction models, but probably because of the short follow-up time and the consequently low event rate the average accuracy of event prediction was only slightly improved compared to ‘prediction’ purely based on the observed event rate. For example, the Brier score without covariates was 0.0092 and only little higher than the values yielded by the models. Likewise, the discrimination slopes indicate that predicted probabilities for persons who later developed a CVD were on average only slightly higher than those who did not develop CVD. The calibration plots were also similar with a slightly better calibration of extreme gradient boosted trees at higher predicted risk (Fig. 3).

At data availabilities of N/10 to N/100, SLNN and GAM achieved better performance measures than MLNN and XGBoost. At N/100, the analytical strategy devised for GAM involved only linear functional relations of predictors with the log odds of the event, which was generally also the case with SLNN. Therefore, these strategies were more stable and less prone to overfitting than others. For example, MLNN yielded particularly poor performance for some subsets at N/100, specifically for the discrimination slope, the AUROC and the AUPRC. Brier scores increased with age as expected (Additional file 2: Appendix 5). However, they did not substantially differ between analytical strategies or data availabilities. The predictive performances at N/2 and N/25 are shown in Additional file 2: Appendix 6.

### Agreement of predictions

At full data availability predictions from all pairs of analytical strategies were highly correlated (≥0.97; Additional file 2: Appendix 7). At N/100, the mean correlation coefficients across the 100 subsets was still high (0.97) for predictions estimated by SLNN and GAM, but the MLNN predictions were quite different from those of the other strategies (mean correlation between 0.65 and 0.84).

### Assessing predictor-risk relation

**Fig. 4:**
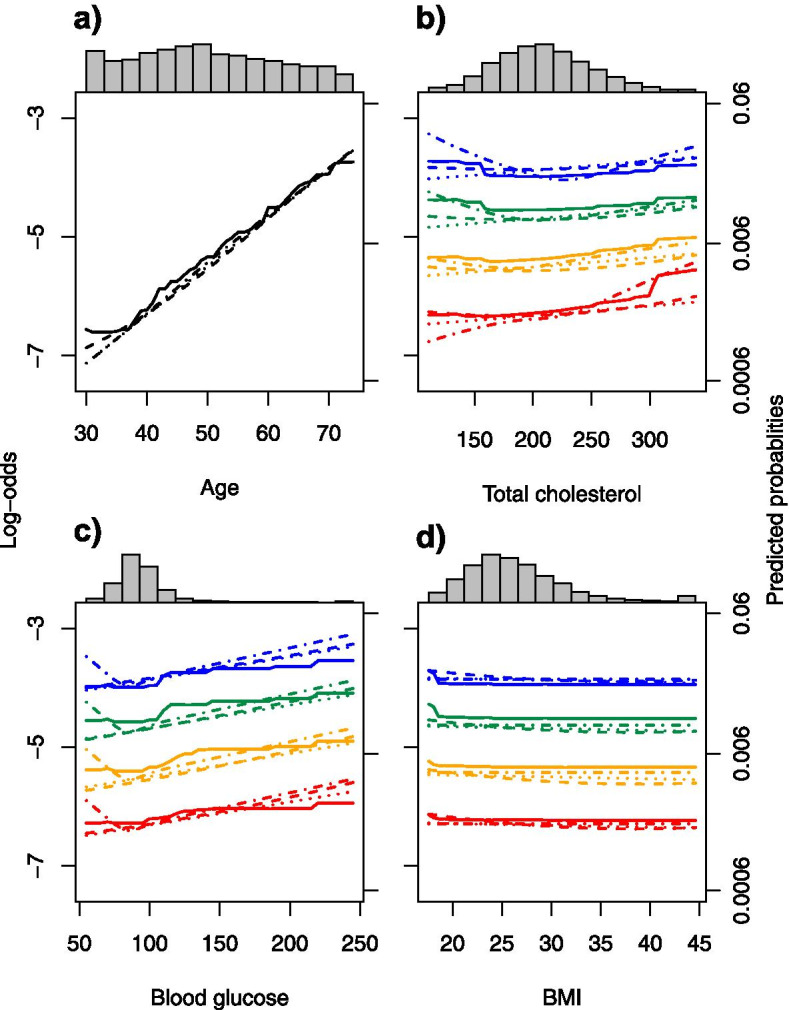
Partial dependence of estimated risk on **a** age, **b** total cholesterol, **c** blood glucose, **d** BMI, showing how average predictions vary with these variables when keeping fixed all other predictors. Black: overall; red: at age fixed at 40 years and sex set to female; yellow: 50 years, female; green: 60 years, female; blue: 70 years, female. Dotted lines: single-layer neural networks/logistic regression; dashed-dotted lines: generalized additive models; dashed lines: multilayer neural networks; solid lines: extreme gradient boosted trees. All models were fitted at full data availability

For assessing and comparing the shape of the estimated predictor-risk relations by means of ICE plots, we focused on the predictors age, total cholesterol, BMI score and blood glucose. For demonstrational purposes we focused on women and for the latter three variables we additionally fixed age at 40, 50, 60 and 70 years. The individual conditional expected risk by age, and the individual expected risk by total cholesterol, BMI and blood glucose conditional on age were parallel for each modeling paradigm at full data availability (Additional file 2: Appendix 8). This justifies averaging of those individual effects for interpretation on a population level with PDPs, which are shown in Fig. 4. By construction, SLNN, GAM and MLNN result in smooth PDPs. XGBoost produced PDPs with steps, which can be explained as resulting from the implicit use of ensembles of classification trees by the algorithm.

The estimated relative log-odds of a cardiovascular event for women were linearly increasing with increasing age for all four analytical strategies (Fig. 4). GAM, MLNN and XGBoost detected a slightly U-shaped effect of total cholesterol for women aged 50, 60 and 70 years. This indicates, e.g., that CVD risk was the lowest with cholesterol values around 200 mg/dL for 60-years old women. However, at 40 years GAM exhibited PDPs indicating an increasing effect. For blood glucose all modeling paradigms identified a linear effect on the predicted log-odds, except for GAM which predicted an increased risk for very low blood glucose levels. Over a wide range of BMI values PDPs were fairly constant indicating no association of BMI with CVD risk. Only XGBoost identified a slightly increased risk with BMIs lower than 18 kg/m^2^, which was unnoticed by other modeling paradigms. Triglycerides and the diastolic blood pressure did not affect CVD risk predictions (Additional file 2: Appendix 9), only XGBoost estimated an increased risk with increasing diastolic blood pressure for 40-year old women. For increasing HDL cholesterol the risk generally decreased as expected. Mens’ PDPs were basically similar to those of women but were slightly shifted upwards towards higher CVD risk (Additional file 2: Appendix 9).

**Fig. 5:**
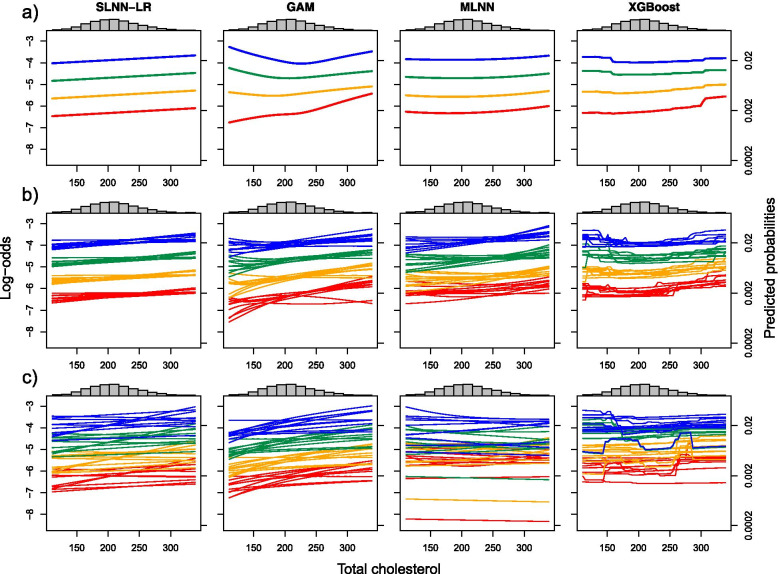
Partial dependence of estimated risk on total cholesterol, showing how average predictions vary with total cholesterol while keeping all other predictors fixed. Red: age fixed at 40 years and sex set to female; yellow: 50 years, female; green: 60 years, female; blue: 70 years, female. The models (SLNN-LR, GAM, MLNN, and XGBoost) were fitted at **a** full data availability **b** data availability of 1/10 and **c** data availability of 1/100. In **c** 10 out of 100 models were randomly selected

Figure 5 compares PDPs for total cholesterol in women between modeling paradigms at different data availabilities. At a data availability of N/10 only XGBoost still reproduced the slightly U-shaped trend of total cholesterol identified at full data availability, however, with less accuracy. The shapes of the PDPs already differed considerably between different subsets, where the highest variability was observed for MLNN. At N/100, SLNN and GAM achieved similar results, whereas MLNN produced highly variable PDPs. Similar comparisons for all data availabilities were done for age, and for total cholesterol, blood glucose and BMI at ages of 40, 50, 60 and 70 years in Additional file 2: Appendix 10–13.

## Discussion

We visualized the relations between individual predictors and the estimated CVD risk that were obtained when model estimation followed different paradigms. We found some similarities between the visualized predictor-risk relations but also occasional divergence. The smaller the sample size, the more the predictor-risk relation depended on the modeling paradigm, and also sampling variability played an increased role, resulting in possibly implausible predictor-risk relations in some models and samples.

The role of ML as new modeling paradigm for prediction in cardiology has been discussed controversially in recent years. While some studies embraced the flexibility of computer-intensive methods which often showed increased predictive performance [27–31], others have pointed at possible pitfalls in comparative studies such as irrelevant increase in predictive power and the focus on discriminative ability while neglecting calibration [13, 32]. It was also pointed out that large data sets are needed to observe a benefit in predictive performance from making prediction rules more complex [33]. Deo and Nallamothu see the future of prediction tasks in ML, but mention a number of limitations, in particular if ML is applied to electronic health records [34]. However, the full potential of standard statistical modeling is likely not exploited as many developments to increase flexibility of statistical models such as modern algorithms for variable selection or for explicitly incorporating non-linear predictor-risk relations in the model structure are still underused [2–4]. The GAM already reaches a certain level of complexity but can still be mathematically described by means of a risk equation (Additional file 3). The other models cannot be written down as a mathematical equation and need implementation in interactive software, e.g. through web access. Some software packages such as R and RStudio allow for straightforward implementation of a web entry form, irrespective of what type of modeling paradigm was used. For models fitted in other software, user access may not be implemented so easily.

Our study is characterized by a number of novel ideas that go beyond simple data analysis. First, we made use of expert knowledge from each modeling culture to develop analytical strategies in a preferred modeling paradigm tailored to the data availabilities, and compared how the different model paradigms induced different interpretations. The multidisciplinary nature of our team with experts from both modeling cultures is a particular strength of our study. Hence, we were able to perform a fair and transparent comparison of analytical strategies. To best mimic the practical situation of analyzing observational studies for predictive research, our study protocol strictly separated the development of the analytical strategies based on the available knowledge at different data availabilities from data analysis. In developing these strategies, background knowledge played different roles. We designed this study to resemble a competition of experts adapting their favorite tools to the modeling question at hand rather than letting a single data analyst compare different default implementations. In this way we have set a new standard for comparison studies.

Our study was characterized by a large sample, with which all analytical strategies led to very similar results with respect to predictive performance. In practice, often less data is available for modeling. Therefore, another feature of our study was the use of simulation to investigate behavior with smaller samples. In particular, we employed subsampling to simulate a more unfavorable events-predictors ratio and demonstrated how our results are affected. With smaller data sets, flexible approaches such as MLNN or XGBoost are at higher risk of overfitting. This is remarkable as in both analytical strategies, overfit was thought to be controlled by reducing the number of predictors at smaller data availabilities (MLNN) or by cross-validation (XGBoost). Another aspect of our study was to demonstrate how the black box of ML models can be opened to explain why a model arrives at a particular result [35]. Lack of explainability of an ML model may be an important obstacle to its bedside use, in particular if consistence with clinical expertise is unclear. Among a variety of tools that were developed to ‘explain’ the predictions of ML models, PDP and ICE plots are attractive as these techniques can also be applied with statistical modeling paradigms such as GAMs, allowing head-to-head comparisons. Some other model-agnostic techniques have been proposed and reviewed recently [36], but were not considered here. XGBoost often produced wiggly PDPs with steps and local peaks that are not rationally interpretable. Our investigation revealed that data availability (sample size) is the decisive factor also for stable and reliable interpretation of the role of predictors in a model. Likewise, it affected the agreement between the predictions from the different analytical strategies – while at high data availabilities, the predictions from all methods were highly correlated, this was no longer the case with smaller but still realistic sample sizes, corroborating the study of Li et al. [37].

Despite these strengths, our study has limitations. First, because of the specific outcome in our study, which occurred in about 1% of the participants in the health screening during the follow-up period of 1 year, we did not compare methods in situations when events are more common. We expect that our results generalize to situations with similar event frequencies, as the number of events is more important than sample size in risk prediction modeling. Moreover, the number of potential predictors for inclusion was limited, and with a higher number of potential predictors, the pre-specification of the complexity allowed in GAMs may become difficult. On the other hand, also ML approaches may suffer from an increased dimensionality of the predictor space, as they must counter overfitting with heavier regularization, and computing time may become an issue as well. We also did not consider using the time-to-event outcome, which may have increased event rates but had brought along new difficulties, as generalizations of SLNN, MLNN and XGBoost suitable for survival-type outcomes are still in their infancy. Lastly, we included only selected analytical strategies in our comparisons, in particular in the field of ML, and ignored some others that are often used in practice such as random forests. Our aim was not to make a wide comparison of ML methods but to use methods that the collaborators in this study were most familiar with, guaranteeing that these methods were employed with appropriate expertise. Moreover, the methods considered here can be considered as representative for novel and more traditional approaches.

The analytical paradigm under which cardiovascular risk prediction models are developed may be largely irrelevant for predictive accuracy of the obtained models and probably also for any conclusions on the role of predictors in these models if certain conditions are met. These conditions comprise a sufficient sample size [38, 39], a flexible enough modeling approach [3], and strict separation of the development of an analytical strategy from data analysis. At lower data availabilities, more flexible approaches are at higher risk of overfitting, which leads to less accurate predictions and less generalizable models. Visualizations such as those exemplified in this work are an indispensable tool to make complex models more transparent and to uncover implausible predictor-risk relations.

## Supplementary Information

**Additional file 1.** TRIPOD checklist.

**Additional file 2.** Extended methods and results.

**Additional file 3.** Risk prediction of a CVD event within 1 year.

## Data Availability

The data underlying this article was provided by the Main Association of Austrian Social Security Institutions (MAASSI) under a cooperation agreement between MAASSI and the Medical University of Vienna. Requests to use this data for research purposes must be addressed to MAASSI at www.sozialversicherung.at.

## Abbreviations

BMI: Body mass index
BP: Blood pressure
CVD: Cardiovascular disease
HDL: High density lipoprotein.
GAM: Generalized additive models
MLNN: Multi-layer neural network
SLNN-LR: Single-layer neural network – logistic regression
XGBoost: Extreme gradient boosted trees
