# Supplementary material for: The roles of predictors in cardiovascular risk models - a question of modeling culture?
Source: BMC Med Res Methodol. 2021 Dec 18;21:284. doi: 10.1186/s12874-021-01487-4 (PMC8684157; doi:10.1186/s12874-021-01487-4)
Supplement: Supplementary file 1 — Additional file 1. TRIPOD checklist. [file 12874_2021_1487_MOESM1_ESM.pdf]

The roles of predictors in cardiovascular risk models - a question of modeling culture?

Authors

Christine Wallisch (1), Asan Agibetov (2), Daniela Dunkler (1), Maria Haller (1,3), Matthias Samwald (2), Georg Dorffner (2), Georg Heinze (1)

Affiliations

(1) Section for Clinical Biometrics, Center for Medical Statistics, Informatics and Intelligent Systems, Medical University of Vienna, Austria
(2) Section for Artificial Intelligence and Decision Support, Center for Medical Statistics, Informatics and Intelligent Systems, Medical University of Vienna, Austria
(3) Ordensklinikum Linz, Hospital Elisabethinen, Department of Nephrology, Linz, Austria

Additional file 1: TRIPOD checklist

TRIPOD Checklist: Prediction Model Development

Section/Topic	Item	Checklist Item	Page
**Title and abstract**			
Title	1	Identify the study as developing and/or validating a multivariable prediction model, the target population, and the outcome to be predicted.	1
Abstract	2	Provide a summary of objectives, study design, setting, participants, sample size, predictors, outcome, statistical analysis, results, and conclusions.	2
**Introduction**			
Background and objectives	3a	Explain the medical context (including whether diagnostic or prognostic) and rationale for developing or validating the multivariable prediction model, including references to existing models.	3-4
	3b	Specify the objectives, including whether the study describes the development or validation of the model or both.	3-4
**Methods**			
Source of data	4a	Describe the study design or source of data (e.g., randomized trial, cohort, or registry data), separately for the development and validation data sets, if applicable.	4-5
	4b	Specify the key study dates, including start of accrual; end of accrual; and, if applicable, end of follow-up.	5
Participants	5a	Specify key elements of the study setting (e.g., primary care, secondary care, general population) including number and location of centres.	5
	5b	Describe eligibility criteria for participants.	5
	5c	Give details of treatments received, if relevant.	-
Outcome	6a	Clearly define the outcome that is predicted by the prediction model, including how and when assessed.	5-6
	6b	Report any actions to blind assessment of the outcome to be predicted.	-
Predictors	7a	Clearly define all predictors used in developing or validating the multivariable prediction model, including how and when they were measured.	6
	7b	Report any actions to blind assessment of predictors for the outcome and other predictors.	-
Sample size	8	Explain how the study size was arrived at.	6-7
Missing data	9	Describe how missing data were handled (e.g., complete-case analysis, single imputation, multiple imputation) with details of any imputation method.	AF 1: Appendix 1
Statistical analysis methods	10a	Describe how predictors were handled in the analyses.	6, AF 1: Appendix 3
	10b	Specify type of model, all model-building procedures (including any predictor selection), and method for internal validation.	7-8, AF 1: Appendix 2
	10d	Specify all measures used to assess model performance and, if relevant, to compare multiple models.	8-9
Risk groups	11	Provide details on how risk groups were created, if done.	
**Results**			
Participants	13a	Describe the flow of participants through the study, including the number of participants with and without the outcome and, if applicable, a summary of the follow-up time. A diagram may be helpful.	Figure 1
	13b	Describe the characteristics of the participants (basic demographics, clinical features, available predictors), including the number of participants with missing data for predictors and outcome.	11
Model development	14a	Specify the number of participants and outcome events in each analysis.	7
	14b	If done, report the unadjusted association between each candidate predictor and outcome.	-
Model specification	15a	Present the full prediction model to allow predictions for individuals (i.e., all regression coefficients, and model intercept or baseline survival at a given time point).	AF 2
	15b	Explain how to the use the prediction model.	AF 2
Model performance	16	Report performance measures (with CIs) for the prediction model.	11-12, Figure 2-3
**Discussion**			
Limitations	18	Discuss any limitations of the study (such as nonrepresentative sample, few events per predictor, missing data).	16-17
Interpretation	19b	Give an overall interpretation of the results, considering objectives, limitations, and results from similar studies, and other relevant evidence.	14-16
Implications	20	Discuss the potential clinical use of the model and implications for future research.	17
**Other information**			
Supplementary information	21	Provide information about the availability of supplementary resources, such as study protocol, Web calculator, and data sets.	Throughout the manuscript
Funding	22	Give the source of funding and the role of the funders for the present study.	18
