# Supplementary material for: The roles of predictors in cardiovascular risk models - a question of modeling culture?
Source: BMC Med Res Methodol. 2021 Dec 18;21:284. doi: 10.1186/s12874-021-01487-4 (PMC8684157; doi:10.1186/s12874-021-01487-4)
Supplement: Supplementary file 2 — Additional file 2. Extended methods and results. [file 12874_2021_1487_MOESM2_ESM.pdf]

The roles of predictors in cardiovascular risk models - a question of modeling culture?

Authors

Christine Wallisch (1), Asan Agibetov (2), Daniela Dunkler (1), Maria Haller (1,3), Matthias Samwald (2), Georg Dorffner (2), Georg Heinze (1)

Affiliations

(1) Section for Clinical Biometrics, Center for Medical Statistics, Informatics and Intelligent Systems, Medical University of Vienna, Austria
(2) Section for Artificial Intelligence and Decision Support, Center for Medical Statistics, Informatics and Intelligent Systems, Medical University of Vienna, Austria
(3) Ordensklinikum Linz, Hospital Elisabethinen, Department of Nephrology, Linz, Austria

Additional file 2: Extended methods and results

Contents

Appendix 1: Preprocessing of the dataset 3

Appendix 2: Overview of analytical strategies applied in each modeling paradigm. 4

Appendix 3 6

a) Detailed analytical strategies of generalized additive models at different data availabilities 6

b) Predictors and degrees of freedom used in the single-layer neural network/logistic regression and in the multi-layer neural network at different data availabilities......................10

c) Hyperparameter settings in gradient boosted trees (XGBoost)...11

Appendix 4: Baseline characteristics of individuals in the training set 13

Appendix 5: Brier score conditional on Age 15

Appendix 6: Predictive performance of the models fitted at full data availability, data availability of N/2, N/10, N/25 and N/100 measured in the test set. 16

Appendix 7: Correlation coefficient for predictions estimated by different modeling paradigms..19

Appendix 8: Estimated predictor-risk relation by individual conditional expectation plots for A) Age, B) Total cholesterol, C) BMI, and D) Blood glucose in 40-, 50-, 60- and 70-year-old women (red, yellow, green, blue) with single-layer neural network/logistic regression (SLNN-LR), generalized additive models (GAM), multi-layer neural networks (MLNN, and extreme gradient boosted trees (XGBoost) fitted at full availability 20

Appendix 9: Estimated predictor-risk relation by partial dependence plots for A) Age, B) Total cholesterol, C) HDL cholesterol, D) Triglycerides, E) Blood glucose, F) Systolic blood pressure, G) Diastolic blood pressure and H) Body mass index in 40-, 50-, 60- and 70-year-old (red, yellow, green, blue) women and men (left, right column) with single-layer neural network/logistic regression (dotted line), generalized additive model (dashed-dotted line), multi-layer neural networks (dashed line), and extreme gradient boosted trees (solid line) fitted at full data availability...21

Appendix 10: Estimated predictor-risk relation by partial dependence plots for age..................26

Appendix 11: Estimated predictor-risk relation by partial dependence plots for total cholesterol 29

Appendix 12: Estimated predictor-risk relation by partial dependence plots for blood glucose...32

Appendix 13: Estimated predictor-risk relation by partial dependence plots for body mass index 35

Appendix 1: Preprocessing of the dataset

Distributions of blood pressure (BP) and blood parameter measurements were truncated at the respective 0.5th and 99.5th percentiles. Missing values in BP treatment (8.4%) were assumed to indicate no treatment. The low number of missing values in risk factors (3.4% of individuals) allowed a complete-case analysis.

Causes of death given in ICD-10 codes (International Classification of Diseases and Related Health Problems, 10th edition) were provided by the Austrian's federal institute for Statistics. Causes of death were split into CVD-related and CVD-unrelated death. For the assignment we used the date of death, gender, and birth year. Unambiguous assignment was possible for 58% of deceased individuals. The remaining deceased individuals, received a probability that his/her death was CVD-related. For an individual, this probability was the number of CVD-related deaths of his/her possible matchings divided by the total number of his/her possible matchings in the registry of deaths. Using these calculated probabilities, CVD-related death was randomly assigned to each deceased individual.

Individuals with a history of CVD were excluded. These events were identified by hospital stays (with information on the discharge diagnoses in ICD-10 codes) prior to the health screening. For discharge diagnoses using ICD-9 codes, we applied forward mapping to transform ICD-9 codes to ICD-10 codes. The ICD-codes for exclusion were I11.0, I13.0, I13.2, I20, I21, I25, I50, I60-I67, I70.2, I73.9 and G45.

Appendix 2: Overview of analytical strategies applied in each modeling paradigm.

This table explains the specific settings chosen for each analytical strategy.

	Modeling paradigm		
	GAM	SLNN-LR & MLNN	XGBoost
Minimized loss function	-2*logLikelihood or cross-entropy	-2*logLikelihood or cross-entropy	-2*logLikelihood or cross-entropy
Background knowledge	Predictors were categorized into 'established' or 'potential'. Established predictors were pre-specified in each model, potential predictors were considered but underwent variable selection.	Two categorized variables (BP classes, BMI classes) were left out assuming they would neither provide much additional information nor would they would be easily be encoded in numerical terms.	-
Pretransformation of variables	Logarithmic transformation of total cholesterol, HDL cholesterol, and cholesterol ratio	Normalization to zero mean and unit variance within each training set	Binary coding of categorical variables
Variable selection	Backward elimination	-	-
Flexibility by	Prespecified interaction terms and restricted cubic splines for continuous variables	Sigmoid hidden units in MLNN	Intrinsic property of the model
Fixed or optimized hyperparameter	Variable selection: *significance level* was 0.157 (which corresponds to the AIC)<br><br>Flexible forms: *number of knots* were fixed to 3 or 4 and the location of knots were set to the default percentile in the function for restricted cubic splines rcs().	The *number of hidden* units was varied according to number of events in the data set.	Four hyperparameters were optimized:<br>*max depth* (upper bound on number of nodes in one tree)<br>*min child weight* (minimum number of observations to split the node)<br>*gamma* (regularization coefficient)<br>*max delta set* (controls update step, useful for imbalanced data sets)<br>The other hyperparameters were set to default (see Supplementary material).
Standard reference	"Regression Modeling Strategies: With Applications to Linear Models, Logistic	„Pattern recognition and machine learning" by Bishop C.M., 2006 [5].	"The Elements of Statistical Learning" by Hastie et al, 2009 (2 edition, corrected in 2017) [21]

	and Ordinal Regression, and Survival Analysis" by Harrell F., 2015 [16]		"Greedy function approximation a gradient boosting machine" by Friedman, 2001 [22]
Program	R version 3.5.0 and package *rms*	Matlab (Mathworks), version 2017, nnet Toolbox	XGboost (v. 0.7.0) with Python (v3.6) bindings

Abbreviations: BMI, body mass index; BP, blood pressure; GAM, generalized additive models; MLNN, multi-layer neural networks; SLNN-LR, single-layer neural network/logistic regression; XGBoost, extreme gradient boosted trees;

Appendix 3

a) Detailed analytical strategies of generalized additive models at different data availabilities

Possible predictors are listed and it is indicated if they were forced into the model or competing for selection by backward elimination with a significance level of 0.157. If the effects of continuous predictors were modelled non-linearly, restricted cubic splines with edf + 1 knots (with default location) were used. Degrees of freedom (df) and effective degrees of freedom (edf) are given. Smaller edf than df are achieved by penalizing the likelihood function (R package *rms*).

	**Full data availability and data availability of N/2**			**Data availability of N/10**			**Data availability of N/25**			**Data availability of N/100**		
(Average) sample size	1,028,739<br>514,370			102,874			41,150			10,287		
(Average) number of events	Women: 3,550; men: 6,220<br>Women: 1,775; men: 3,110			Women: 355 ; Men: 622			391			98		
**Predictors**	F / S*	Non-linear?	df/edf	F / S*	Non-linear?	df/edf	F / S*	Non-linear?	df/edf	F / S*	Non-linear?	df/edf
Sex	Separate models			Separate models			F	Yes	1/1	F		1/1
Age	F	Yes	2/2	F	Yes	2/2	F	Yes	2/2	F		1/1
log(Cholesterol ratio)				F	Yes	3/3	F	Yes	3/3	F		1/1
log(Total cholesterol)	F	Yes	3/3									
log(HDL cholesterol)	F	Yes	3/3									
Systolic BP	F	Yes	3/3	F	Yes	3/3	F	Yes	3/3	F		1/1
Hypertensive drug intake	F	Yes	1/1	F		1/1	F		1/1	F		1/1
Smoker	F	Yes	1/1	F		1/1	F		1/1	F		1/1
Diabetic	F	Yes	1/1	F		1/1	F		1/1	F		1/1
BMI	S	Yes	3/3	S	Yes	3/3	S	Yes	3/3			
Waist size	S		1/1	S		1/1						
Physical activity	S		2/2	S	Yes	2/2						
Diastolic blood pressure	S	Yes	3/3									
Blood sugar level	S	Yes	3/3									
Triglycerides	S	Yes	3/3									
Glucose in urine	S		1/1									
Protein in urine	S		1/1	S	Yes	1/1	S		1/1			
**Interaction terms**												

Systolic BP * Hypertensive drug intake	F	Yes	3/3	F	Yes	3/3	F	Yes	3/1	F		1/0.3
Age*log(Total cholesterol)	F	Yes	6/6									
Age*log(HDL cholesterol)	F	Yes	6/6									
Age*log(Cholesterol ratio)				F	Yes	6/6	F	Yes	6/2	F		1/0.3
Age*Smoker	F	Yes	2/2	F	Yes	2/2	F	Yes	2/0.7	F		1/0.3
Age*Diabetic	F	Yes	2/2	F	Yes	2/2	F	Yes	2/0.7	F		1/0.3
Diastolic BP * Hypertensive drug intake	S	Yes	3/3									
Blood sugar level * Diabetic	S	Yes	3/3									
Sex*log(Cholesterol ratio)							F	Yes	3/0.7	F		1/0.3
Sex*Smoker							F		1/0.3	F		1/0.3
Sex*Diabetic							F		1/0.3	F		1/0.3
Total edf			56			31			21.7			10
Edof			Women: 63; Men: 111<br>Women: 32; Men: 56			Women: 11; Men: 20			18			10
Required sample size according to recommendations of Riley et al. (2020)[**]			Women: 15,677; Men: 8,153			Women: 4,514; Men: 8,678			3,484			1,936

Abbreviations: BMI, body mass index; BP, blood pressure; df, degrees of freedom; edf, effective degrees of freedom; edof, events per degree of freedom; HDL, high-density lipoprotein

[*] F = forced into the model, S = competing for selection;

[**] **Calculation of required sample size**

Sample size calculations were performed for each data availability considering performance measures from existing literature for the assumptions of this calculation.

We assumed a level of shrinkage of 0.9 and a Cox-Snell R-squared of 0.05, 0.1 and 0.075 for women, men and both, respectively. We used the website https://riskcalc.org/pmsamplesize/, which is based on recommendations of Riley et al. (2020),[25] to calculate the required sample size with observed outcome rates of 0.006, 0.013 and 0.009 in women, in men and overall:

Appendix 3a.1: Sample size calculations for generalized additive models

Sex	Outcome rate	Cox-Snell R²	Level of shrinkage	**Calculated sample sizes**			
				**Full data availability & data availability of N/2**	**Data availability of N/10**	**Data availability of N/25**	**Data availability of N/100**
				56 candidate predictors (edof)	31 candidate predictors (edof)	18 candidate predictors (edof)	10 candidate predictors (edof)
Women	0.006	0.050	0.9	15,677	8,678		
Men	0.013	0.100	0.9	8,153	4,514		
Overall	0.009	0.075	0.9			3,484	1,936

Derivation of Cox-Snell R² values from c-statistics

For the calculation of Cox-Snell R², we extracted c-statistics of 0.79 and 0.76 for women and men from D'Agostino et al. (2008), who designed a prediction model for 10-year CVD risk (Framingham model). [1] Then we rounded them down to 0.78 and 0.76 which were available in the conversion table to Cox-Snell R². The corresponding Cox-Snell R² were 0.076 and 0.118 for women and men (see calculation below). We assumed that with a follow-up of 1 year, slightly lower values must be expected which we assumed as 0.05 and 0.10, respectively. For models including both sexes, we assumed a Cox-Snell R² of 0.075.

Calculation of Cox-Snell R-squared from the c statistics (or AUROC) followed the explanations given by Riley et al. (2019):[36]

- Extract R_D^2 from table 2 of Riley et al. (2019) for the corresponding c statistics.
- Use R_D^2 as a proxy for $R^2_{Royston\_app}$ and compute $R^2_{O'Quigley\_app} = \frac{-\frac{\pi^2}{6} R^2_{Royston\_app}}{\left(1-\frac{\pi^2}{6}\right) R^2_{Royston\_app} - 1}$.
- Calculate $LR = -E \ln\left(1 - R^2_{O'Quigley\_app}\right)$ whereby E is the number of events
- Compute $R^2_{CS\_app} = 1 - \exp\left(-\frac{LR}{n}\right)$.
- Compute $S_{VH} = 1 + \frac{p}{n \ln\left(1-R^2_{CS\_app}\right)}$ whereby n is the sample size used for training and p is the number of candidate predictors.
- Finally, calculate $R^2_{CS\_adj} = S_{VH} R^2_{CS\_app}$.

Below one finds all intermediate results obtained in the calculation of the Cox-Snell R^2 based on information (n, E, p, c-statistics) from the Framingham model:

<u>Appendix 3b.2: Intermediate results for sample size calculation for generalized additive models</u>

Sex	n	E	p	c statistic	R_D^2 ($\sim R_{Royston}^2$)	$R_{O'Quigley\_app}^2$	LR	$R_{CS\_app}^2$	S_{VH}	$R_{CS\_adj}^2$
Women	4522	456	7	0.78	0.427	0.551	364.854	0.078	0.981	0.076
Men	3969	718	7	0.76	0.382	0.504	503.676	0.119	0.986	0.118

b) Predictors and degrees of freedom used in the single-layer neural network/logistic regression and in the multi-layer neural network at different data availabilities

Possible predictors are listed. Degrees of freedom (df) and effective degrees of freedom (edf) are given for the MLP.

	**Full data availability**	**Data availability of N/2**	**Data availability of N/10**	**Data availability of N/25**	**Data availability of N/100**
(Average) sample size	1,028,739	514,370	102,874	41,150	10,287
(Average) number of events	9,770	4,885	977	391	98
**Predictors included**					
Sex	Yes	Yes	Yes	Yes	Yes
Age	Yes	Yes	Yes	Yes	Yes
Cholesterol ratio	Yes	Yes	Yes	Yes	Yes
Total cholesterol	Yes	Yes	Yes	Yes	Yes
HDL cholesterol	Yes	Yes	Yes	Yes	Yes
Systolic BP	Yes	Yes	Yes	Yes	Yes
Hypertensive drug intake	Yes	Yes	Yes	Yes	Yes
Smoker	Yes	Yes	Yes	Yes	Yes
Diabetic	Yes	Yes	Yes	Yes	Yes
BMI	Yes	Yes	Yes	Yes	
Waist size	Yes	Yes	Yes	Yes	
Physical activity	Yes	Yes	Yes	Yes	
Diastolic blood pressure	Yes	Yes	Yes	Yes	
Blood sugar level	Yes	Yes	Yes	Yes	
Triglycerides	Yes	Yes	Yes	Yes	
Glucose in urine	Yes	Yes	Yes	Yes	
Protein in urine	Yes	Yes	Yes	Yes	
Number of hidden units	30	25	5	3	2
Total df (MLP)	540	450	90	54	18
Edof	19	11	11	7.2	5.4

Abbreviations: BMI, body mass index; BP, blood pressure; df, degrees of freedom; edof, events per degree of freedom; HDL, high-density lipoprotein; MLP: multilayer perceptron

c) Hyperparameter settings in gradient boosted trees (XGBoost)

The list of all XGBoost hyperparameters, their meaning, and the recommended search space are detailed in the documentation page (https://xgboost.readthedocs.io/en/latest/parameter.html). We started with the default parameters for all data subsets.

```
{'base_score': 0.5,
 'booster': 'gbtree',
 'colsample_bylevel': 1,
 'colsample_bytree': 1,
 'gamma': 0,
 'learning_rate': 0.1,
 'max_delta_step': 0,
 'max_depth': 3,
 'min_child_weight': 1,
 'missing': None,
 'n_estimators': 100,
 'nthread': 1,
 'objective': 'binary:logistic',
 'reg_alpha': 0,
 'reg_lambda': 1,
 'scale_pos_weight': 1,
 'seed': 0,
 'silent': 1,
 'subsample': 1}
```

Then, for each model at each data availability, we performed a grid search on four parameters in a 5-fold cross-validation. These were the exact values we allowed in the grid search:

```
{
    'gamma': [0, 1, 5],
    'max_depth': [3, 6, 8],
    'max_delta_step': [0, 1, 5],
    'min_child_weight': [1, 5],
}
```

To determine optimal parameters we compared the area under the receiver operating characteristic curve (AUROC) on validation sets (splits from the training data). Below we compare AUROC of optimized and default XGBoost models:

Supplementary Side Table 2c.1: Optimized and default parameters of XGBoost models

data availability	default parameters	optimized
1/1	0.8 +/- 0.3%	0.8 +/- 0.3%
1/2	0.8 +/- 0.3%	0.8 +/- 0.3%
1/10	0.79 +/- 0.2%	0.8 +/- 0.3%
1/25	0.77 +/- 1%	0.79 +/- 0.8%
1/100	0.72 +/- 3%	0.78 +/- 2%

Models based on lower data availabilities benefited the most from the optimization. Optimization had little effect on the accuracy for models based on full data availability and data availability at N/2.

Finally, we show mean values of the optimized parameters at each data availability. As we can see, models based on lower data availabilities (N/25, N100) had deeper trees, however were regularized harsher (higher gamma values). The chosen parameters may indicate a possible overfitting behavior of these models.

Supplementary Side Table 2c.2: Mean value of the optimized parameters of XGBoost models

data availability	gamma	max_depth	max_delta_step	min_child_weight
1/1	0 (0)	3 (0)	0 (0)	1 (0)
1/2	0 (0)	3 (0)	0 (0)	1 (0)
1/10	2.3 (2.35)	3 (0)	1 (0)	2.6 (2)
1/25	2.6 (2.1)	4 (1.84)	1.28 (1.13)	3.56 (1.95)
1/100	3.62 (2)	4.92 (2)	2.23 (2)	2.88 (2)

Models based on full data availability or N/2 with default parameters were performing best. For lower data availabilities we provide mean (SD) of parameter values for all models in the data subset.

Appendix 4: Baseline characteristics of individuals in the training set

	Training set (n=1,028,739)	
	**Men (n=475379; 46.2%)**	**Women (n=553360; 53.8%)**
**Age (years)**	49.9 (11.9)	50.1 (12.2)
**Total cholesterol (mg/dl)**	210 (42.0)	212 (41.6)
**HDL cholesterol (mg/dl)**	51.4 (14.9)	64.0 (17.2)
**Cholesterol ratio (total cholesterol/HDL cholesterol)**	4.38 (1.43)	3.54 (1.14)
**Triglycerides (mg/dl)**	146 (94.9)	109 (62.5)
**Blood glucose (mg/dl)**	98.2 (24.8)	92.7 (20.3)
**Systolic BP (mmHg)**	133 (17.2)	127 (18.7)
**Diastolic BP (mmHg)**	82.7 (10.1)	79.6 (10.4)
**BP classes**		
Ideal	51,568 (10.8%)	134,764 (24.4%)
Normal	120,077 (25.3%)	149,163 (27.0%)
Still normal	111,942 (23.5%)	104,459 (18.9%)
Hypertension stage 1	112,270 (23.6%)	90,479 (16.4%)
Hypertension stage 2	14,414 (3.0%)	12,537 (2.3%)
Isolated systolic hypertension	65,108 (13.7%)	61,958 (11.2%)
**Hypertensive drug intake**		
Yes (vs. no)	66,300 (13.9%)	69,223 (12.5%)
**Smoking status**		
Yes (vs. no)	118,334 (24.9%)	115,935 (21.0%)
**Diabetes**		
Yes (vs. no)	27,337 (5.8%)	21,852 (3.9%)
**BMI score (kg/m²)**	27.4 (4.69)	26.0 (5.67)
**BMI classes**		
<18.5	1,816 (0.4%)	13,180 (2.4%)
18.5-24.9	151,366 (31.8%)	273,342 (49.4%)
25.0-29.9	218,323 (45.9%)	157,092 (28.4%)
30.0-34.9	74,541 (15.7%)	68,474 (12.4%)
35.0-39.9	16,383 (3.4%)	22,376 (4.0%)
≥40.0	12,950 (2.7%)	18,896 (3.4%)
**Waist circumference**		
Too large (vs. okay)	166,486 (35.0%)	191,717 (34.6%)

	**Training set (n=1,028,739)**	
	**Men (n=475379; 46.2%)**	**Women (n=553360; 53.8%)**
**Physical activity**		
None	52,766 (11.1%)	62,373 (11.3%)
Ocasional	202,770 (42.7%)	236,868 (42.8%)
Regular	219,843 (46.2%)	254,119 (45.9%)
**Protein in urine**		
Positive (vs. negative)	28,738 (6.0%)	32,513 (5.9%)
**Glucose in urine**		
Positive (vs. negative)	10,926 (2.3%)	8,603 (1.6%)

Continuous variables are reported as mean (sd) and for categorical variables absolute numbers and percentages are given.

Abbreviations: BMI, body mass index, BP, blood pressure; HDL, high density lipoprotein.

Appendix 5: Brier score conditional on Age

Brier score as a function of age for the model paradigms (SLLN-LR, GAM, MLNN, XGBoost) trained at full data availability, data availability of N/2, N/10, N/25 and N/100, evaluated in the test set.

Abbreviations: GAM, generalized additive models; MLNN, multi-layer neural networks; SLNN-LR, single-layer neural network/logistic regression; XGBoost, extreme gradient boosted trees;

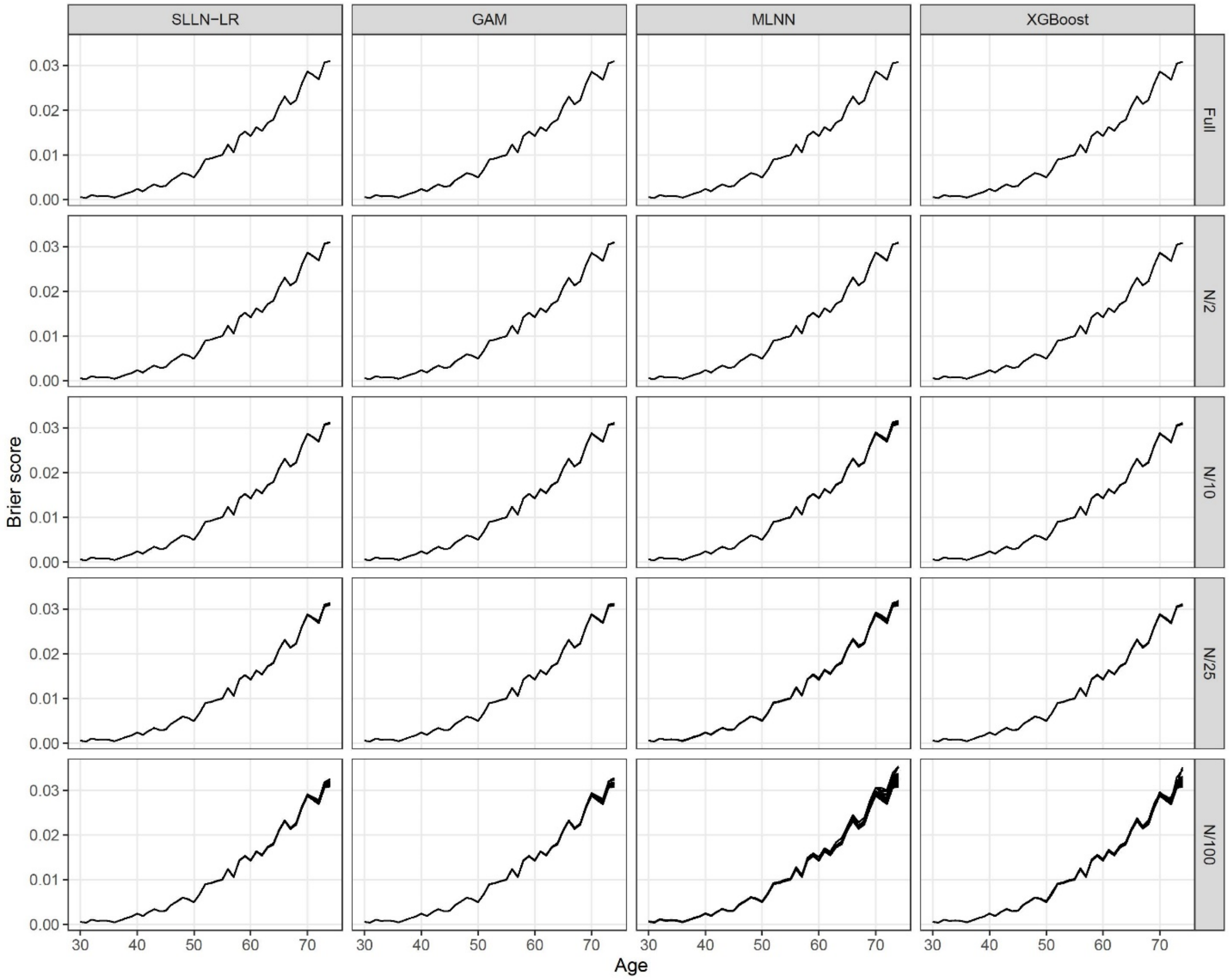

Appendix 6: Predictive performance of the models fitted at full data availability, data availability of N/2, N/10, N/25 and N/100 measured in the test set.

Brier Score, discrimination slope, AUROC, AUPRC and calibration plot for single-layer neural network/logistic regression (red) generalized additive models (rose), multi-layer neural network (violet) and extreme gradient boosted trees (blue), evaluated in the test set.

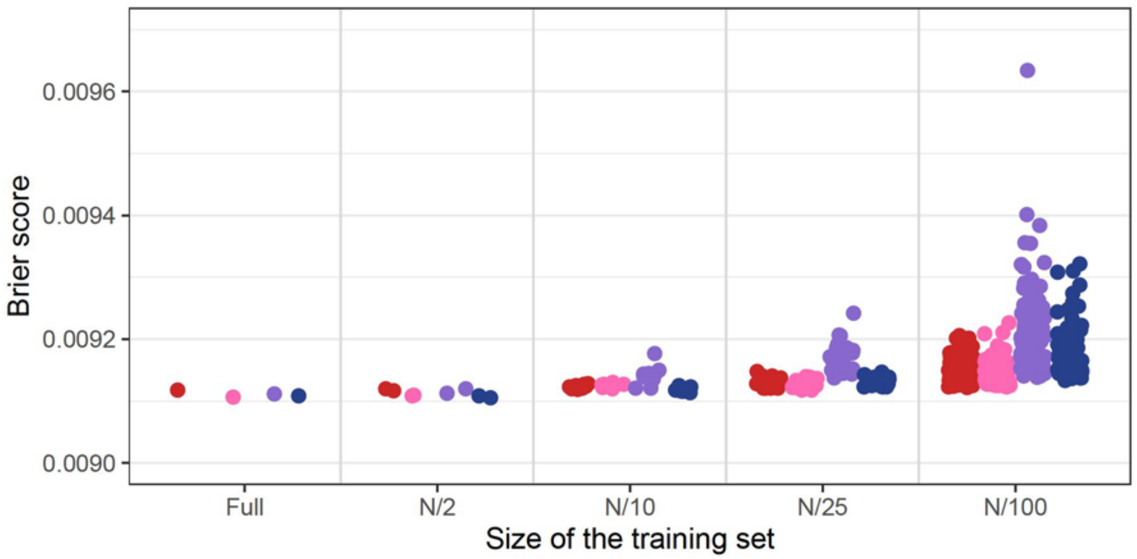

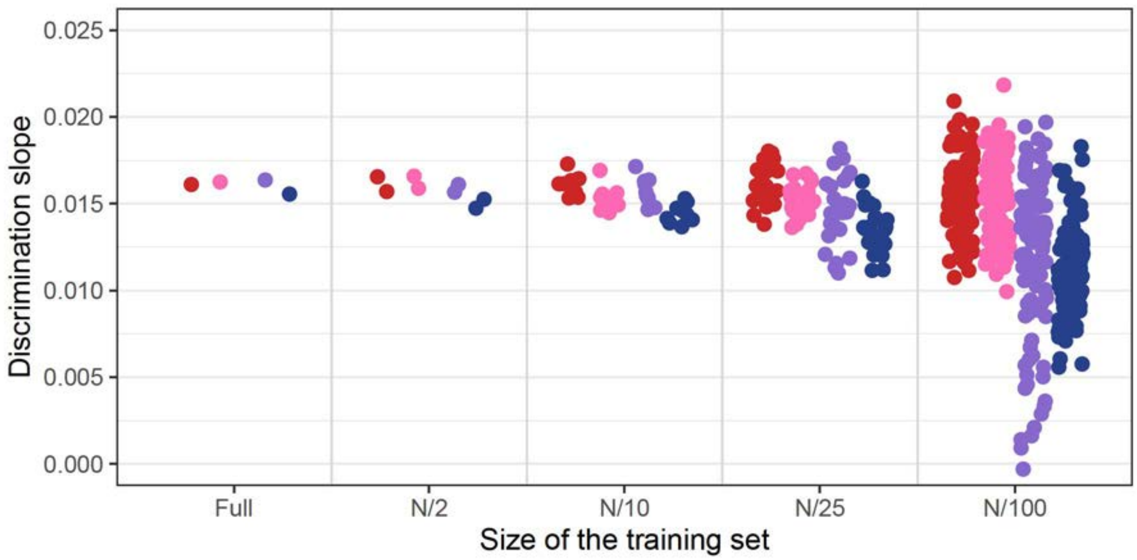

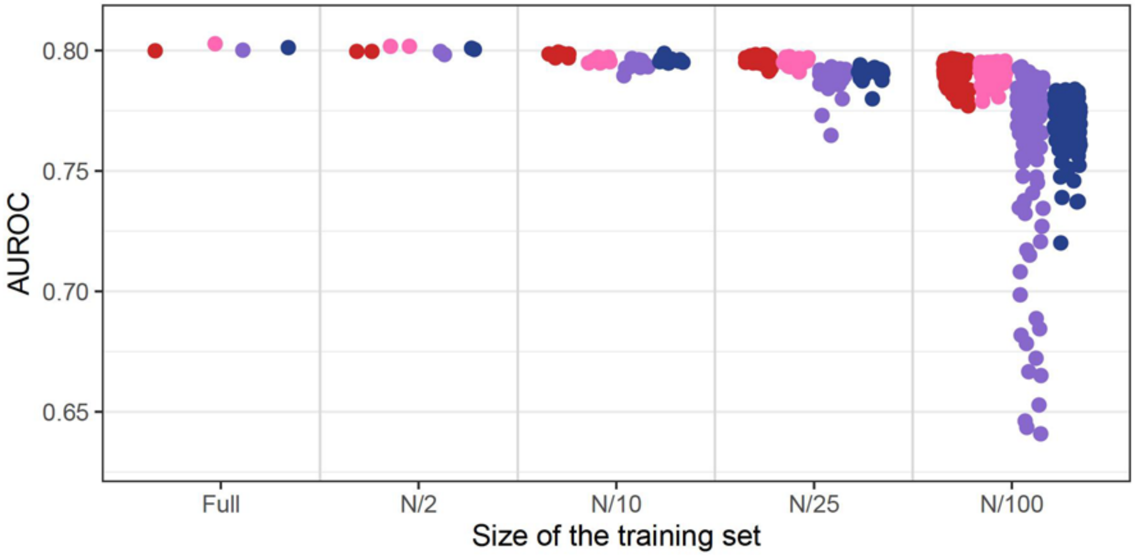
0.80
0.75
0.70
0.65
AUROC
Full
N/2
N/10
N/25
N/100
Size of the training set

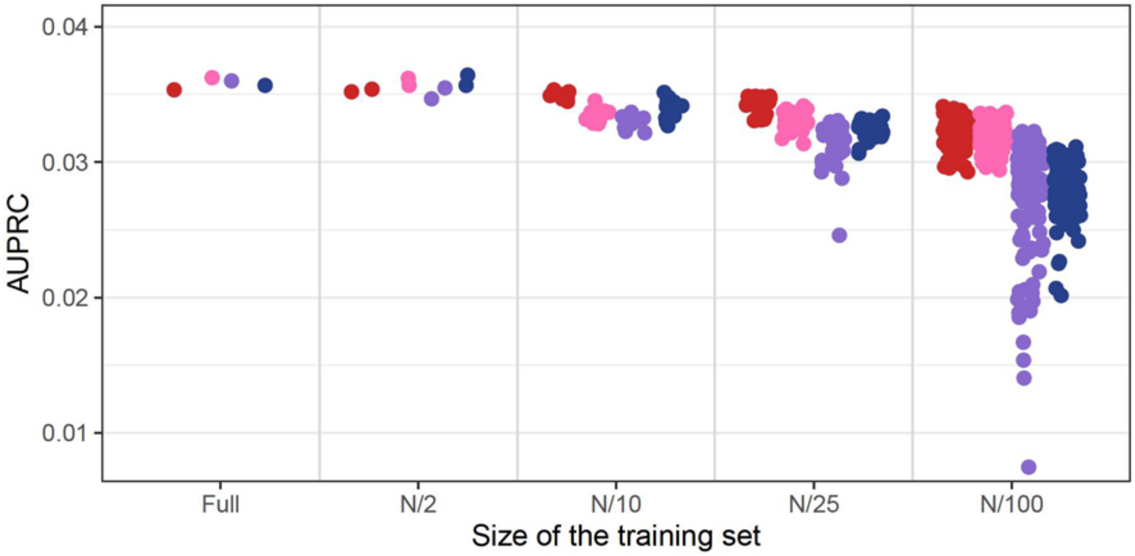
0.04
0.03
0.02
0.01
AUPRC
Full
N/2
N/10
N/25
N/100
Size of the training set

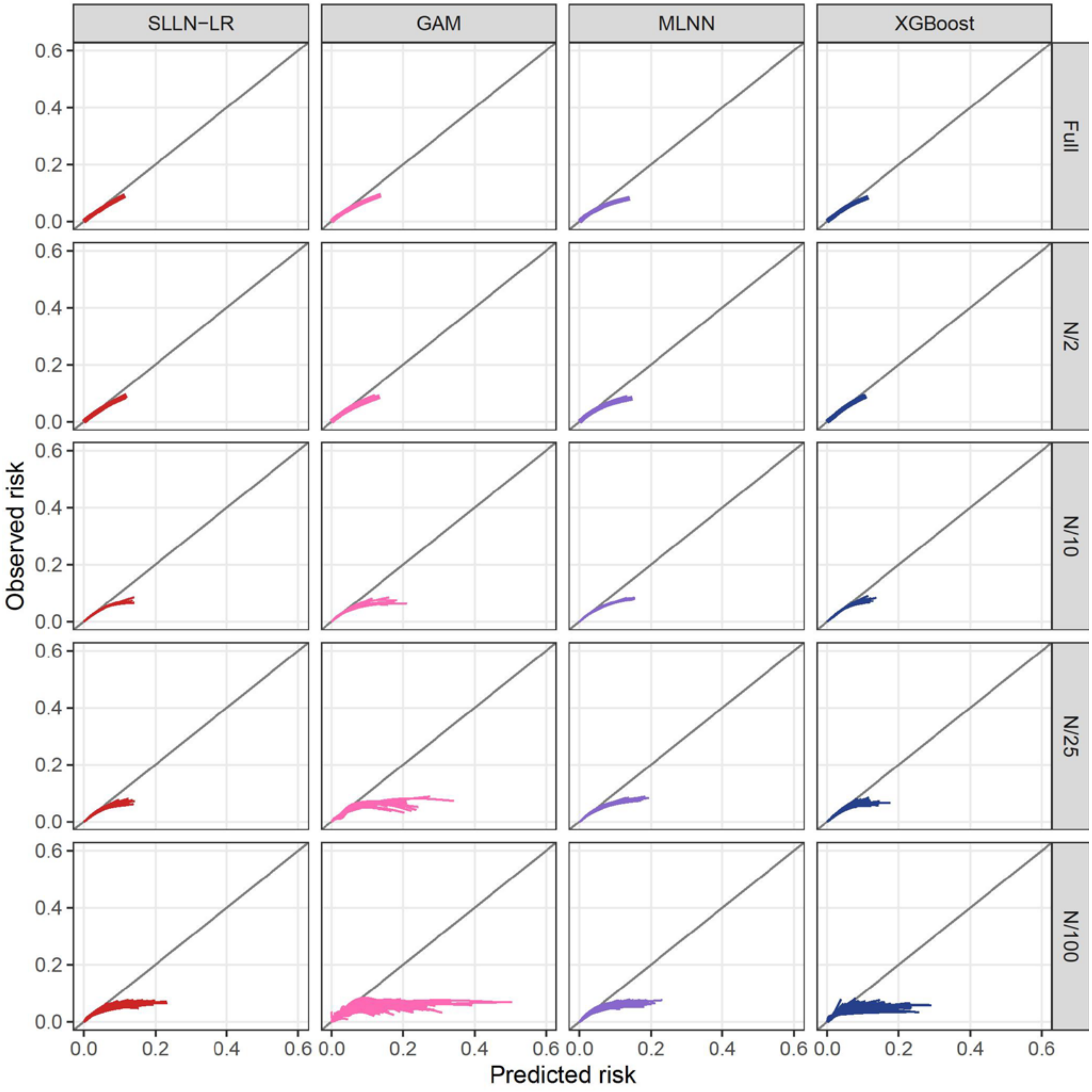
SLLN−LR
GAM
MLNN
XGBoost
Full
N/2
N/10
N/25
N/100
Observed risk
Predicted risk
0.0
0.2
0.4
0.6

Appendix 7: Correlation coefficient for predictions estimated by different modeling paradigms

(Mean) Spearman's rank correlation coefficient of predictions between different modeling paradigms (SLLN-LR, GAM, MLNN, XGBoost) trained at full data availability, data availability of N/10 and N/100, evaluated in the test set. The correlation was averaged over the models derived at data availability of 1/10 and 1/100.

Abbreviations: GAM, generalized additive models; MLNN, multi-layer neural networks; SLNN-LR, single-layer neural network/logistic regression; XGBoost, extreme gradient boosted trees;

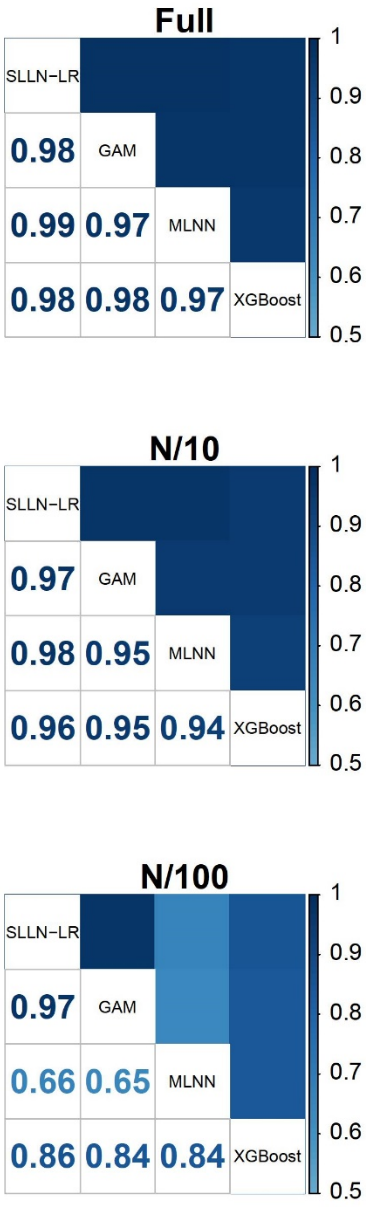

Appendix 8: Estimated predictor-risk relation by individual conditional expectation plots for A) Age, B) Total cholesterol, C) BMI, and D) Blood glucose

in 40-, 50-, 60- and 70-year-old women (red, yellow, green, blue) with single-layer neural network/logistic regression (SLNN-LR), generalized additive models (GAM), multi-layer neural networks (MLNN, and extreme gradient boosted trees (XGBoost) fitted at full availability

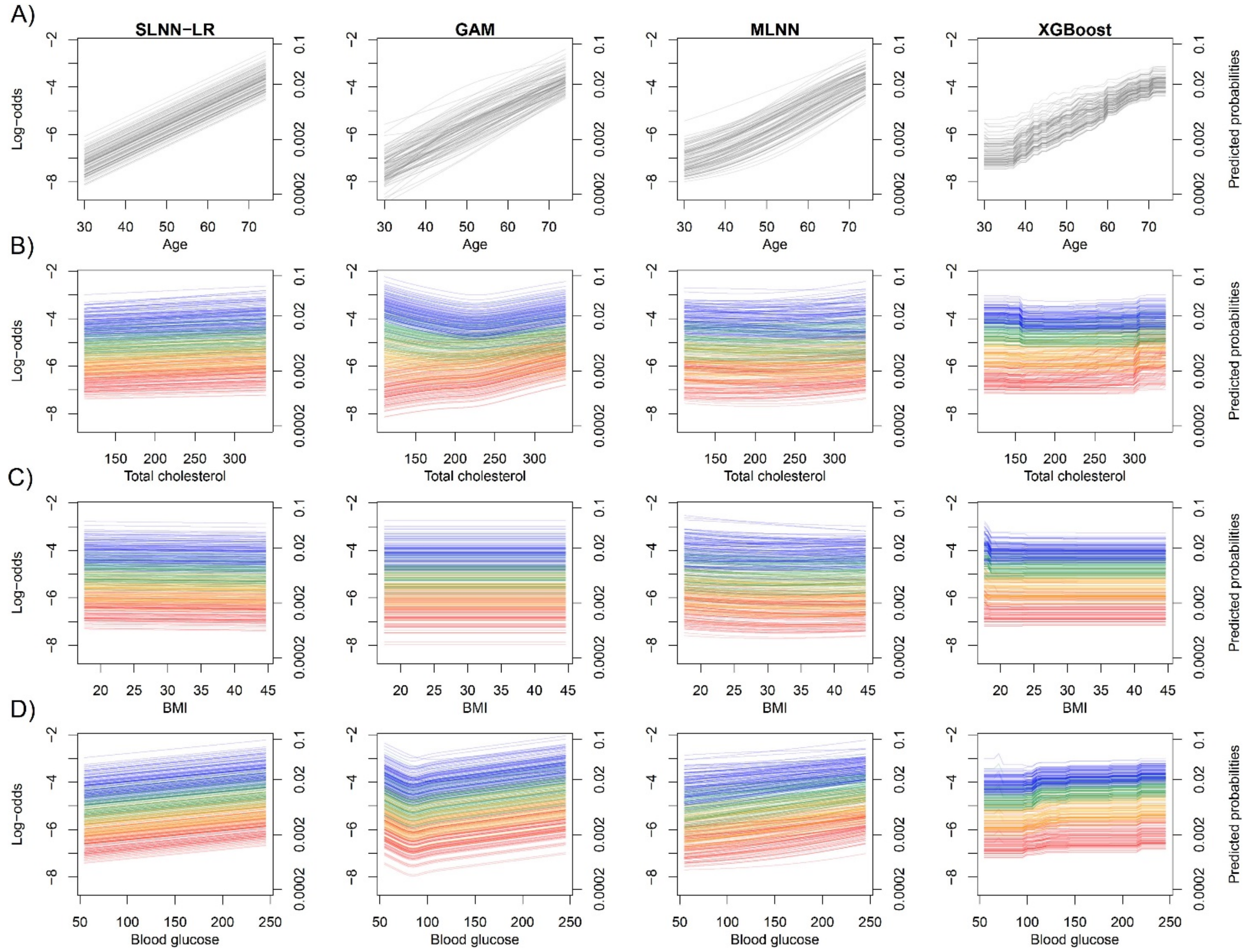

Abbreviations: BMI, body mass index

Appendix 9: Estimated predictor-risk relation by partial dependence plots for A) Age, B) Total cholesterol, C) HDL cholesterol, D) Triglycerides, E) Blood glucose, F) Systolic blood pressure, G) Diastolic blood pressure and H) Body mass index

in 40-, 50-, 60- and 70-year-old (red, yellow, green, blue) women and men (left, right column) with single-layer neural network/logistic regression (dotted line), generalized additive model (dashed-dotted line), multi-layer neural networks (dashed line), and extreme gradient boosted trees (solid line) fitted at full data availability.

Women **Men**

A)

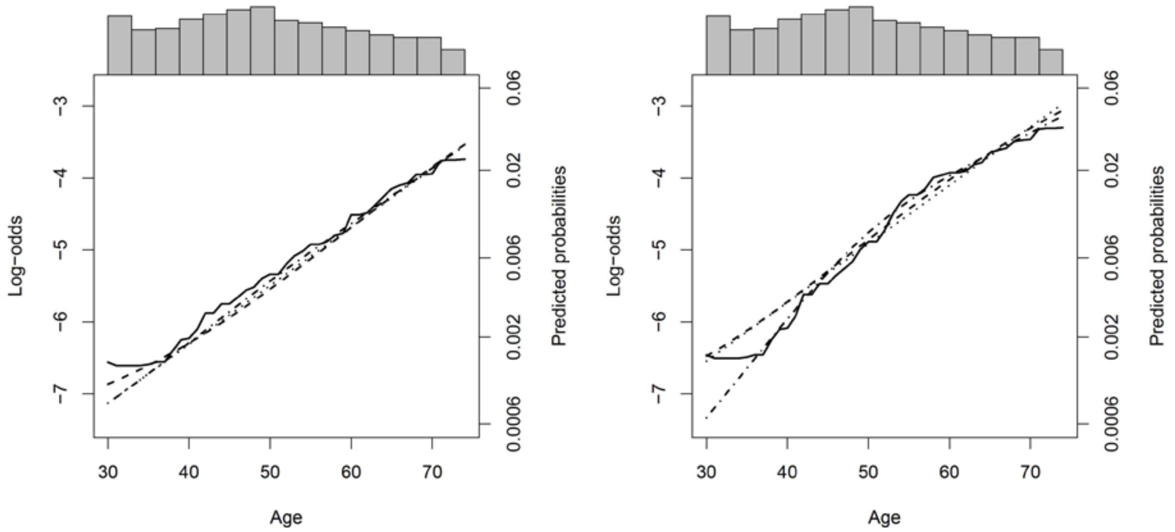

B)

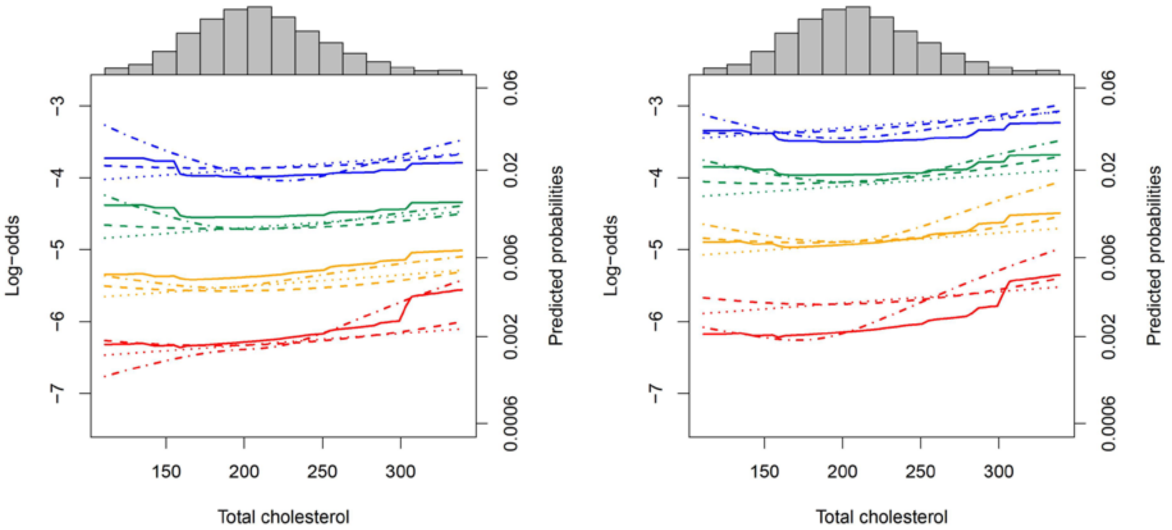
Log−odds
Predicted probabilities
Total cholesterol
Log−odds
Predicted probabilities
Total cholesterol

C)

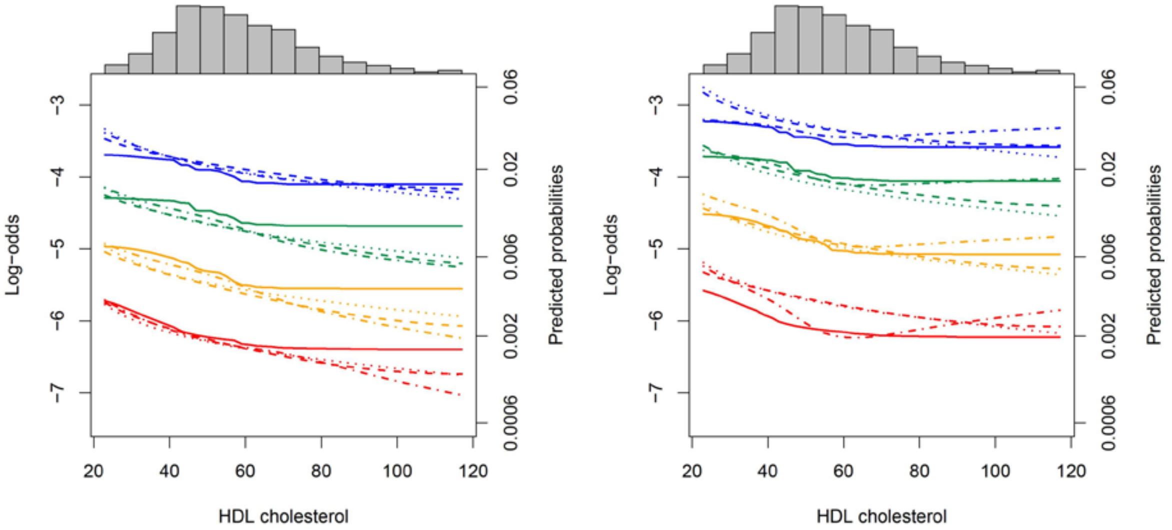
Log−odds
Predicted probabilities
HDL cholesterol
Log−odds
Predicted probabilities
HDL cholesterol

D)

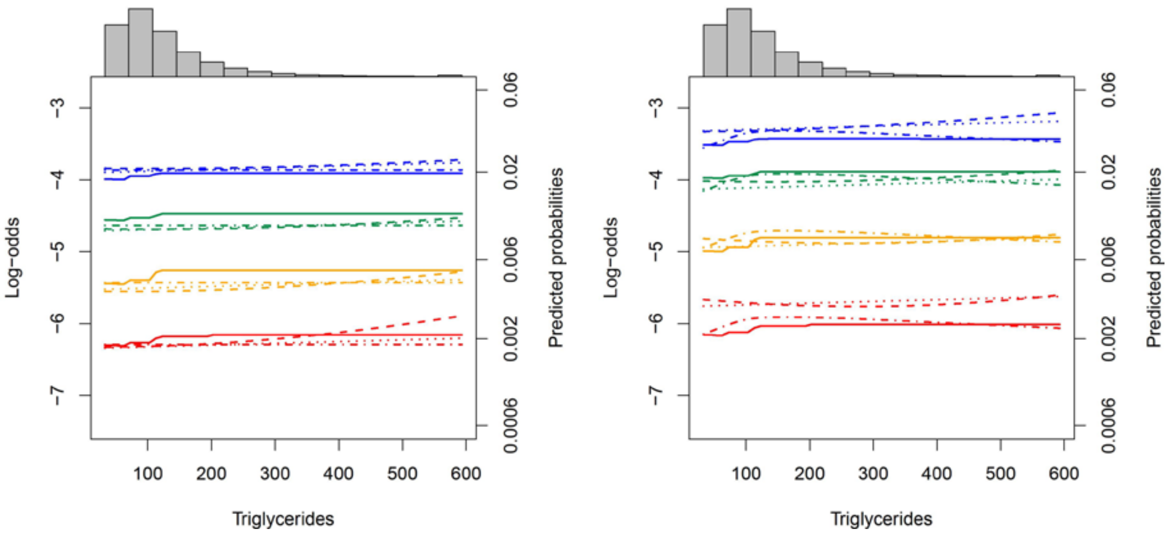

−3
−4
−5
−6
−7
Log−odds
0.06
0.02
0.006
0.002
0.0006
Predicted probabilities
100 200 300 400 500 600
Triglycerides
−3
−4
−5
−6
−7
Log−odds
0.06
0.02
0.006
0.002
0.0006
Predicted probabilities
100 200 300 400 500 600
Triglycerides

E)

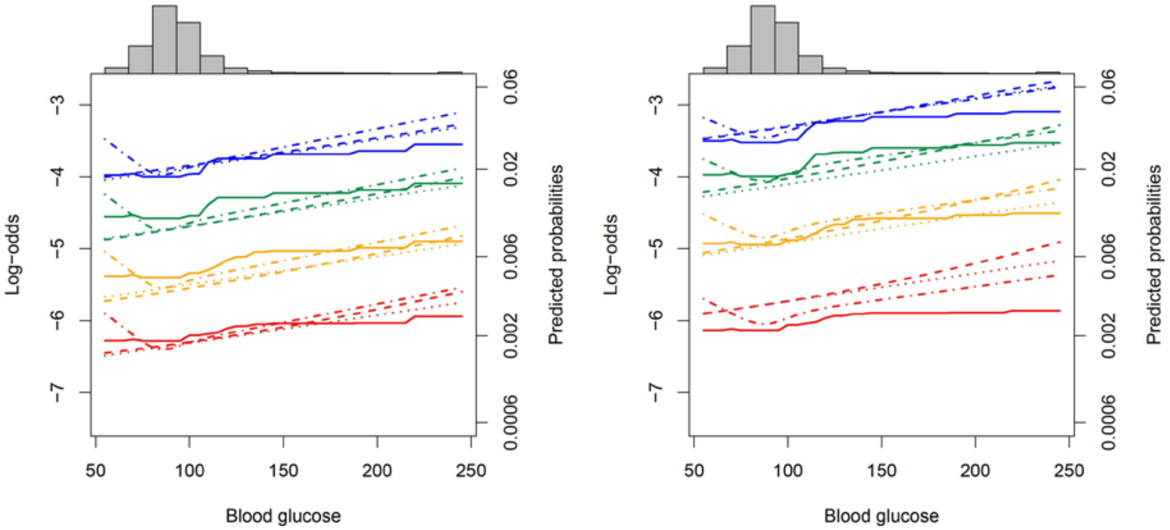

−3
−4
−5
−6
−7
Log−odds
0.06
0.02
0.006
0.002
0.0006
Predicted probabilities
50 100 150 200 250
Blood glucose
−3
−4
−5
−6
−7
Log−odds
0.06
0.02
0.006
0.002
0.0006
Predicted probabilities
50 100 150 200 250
Blood glucose

F)

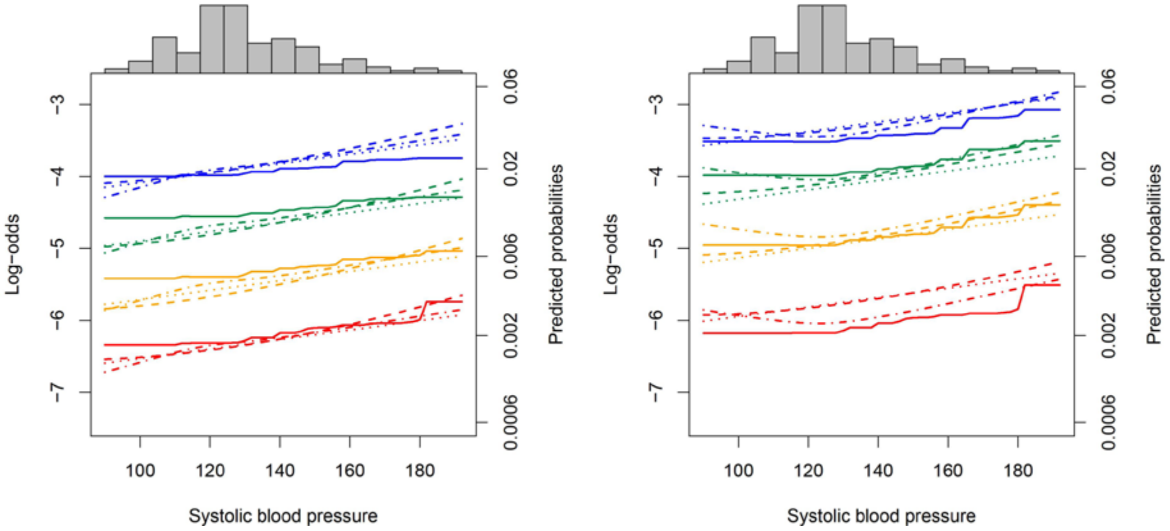
Log−odds
Predicted probabilities
Systolic blood pressure
−3
−4
−5
−6
−7
0.06
0.02
0.006
0.002
0.0006
100
120
140
160
180

G)

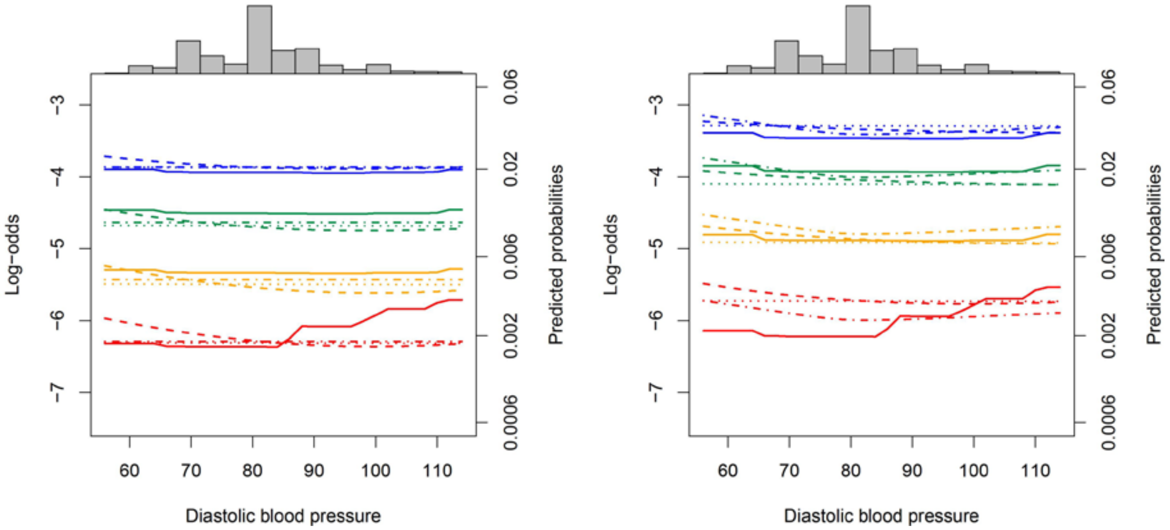
Log−odds
Predicted probabilities
Diastolic blood pressure
−3
−4
−5
−6
−7
0.06
0.02
0.006
0.002
0.0006
60
70
80
90
100
110

H)

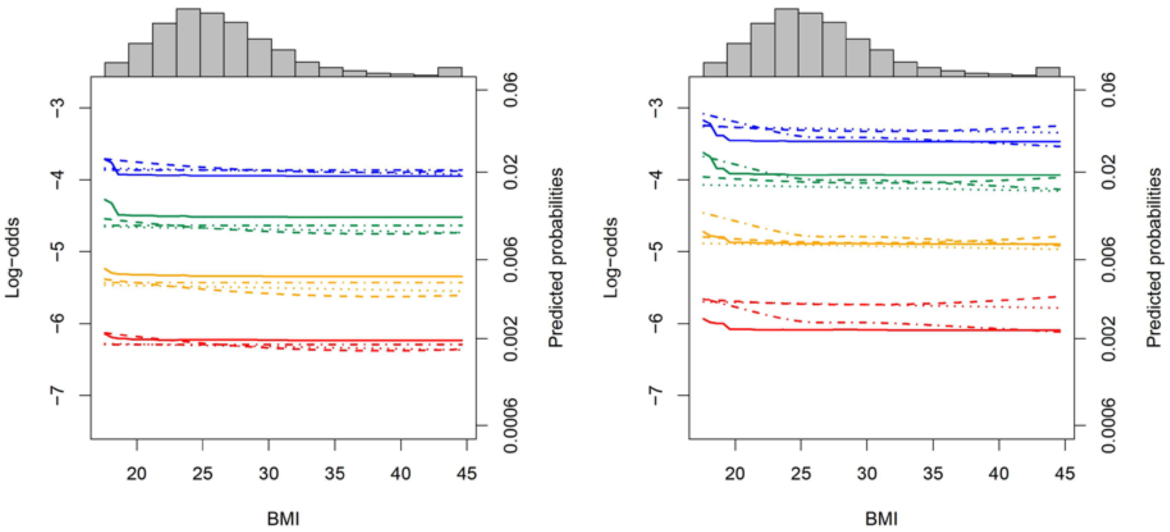

Log−odds
Predicted probabilities
BMI
Log−odds
Predicted probabilities
BMI

Appendix 10: Estimated predictor-risk relation by partial dependence plots for age

Partial dependence plots for Age in women with all modeling paradigms (SLNN-LR, GAM, MLNN, XGBoost) fitted at A) full data availability, B) data availability of N/2, C) data availability of N/10, D) data availability of N/25 and E) data availability of N/100. In D) and E) ten out of 25 or 100 random models were selected.

Abbreviations: GAM, generalized additive models; MLNN, multi-layer neural networks; SLNN-LR, single-layer neural network/logistic regression; XGBoost, extreme gradient boosted trees;

A)

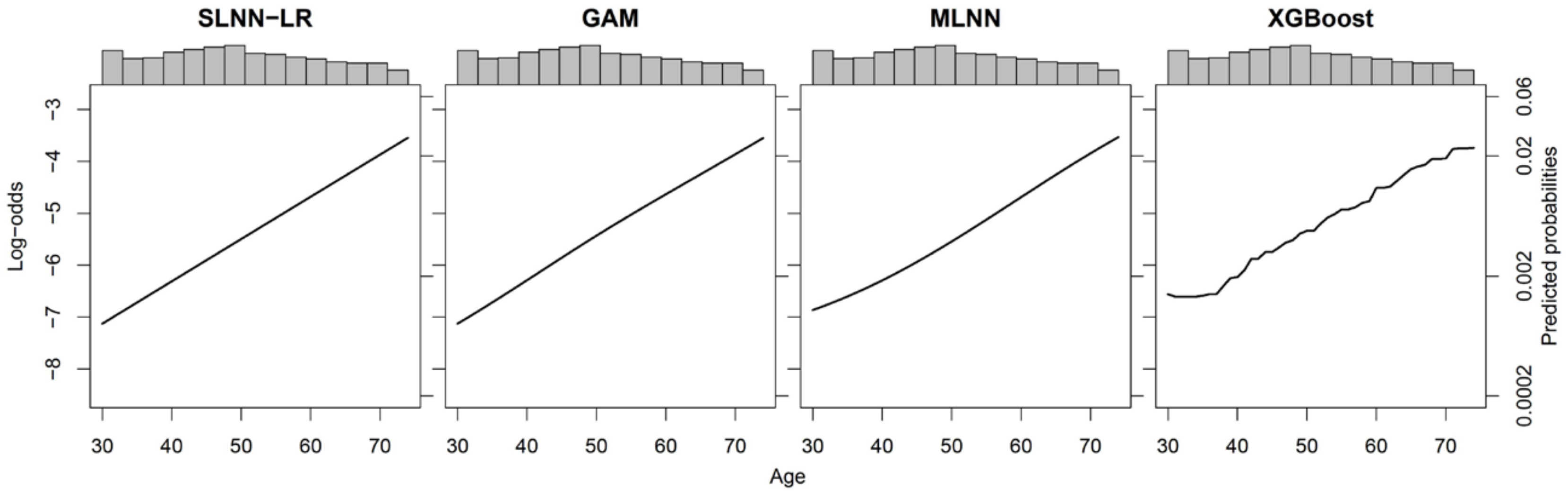

B)

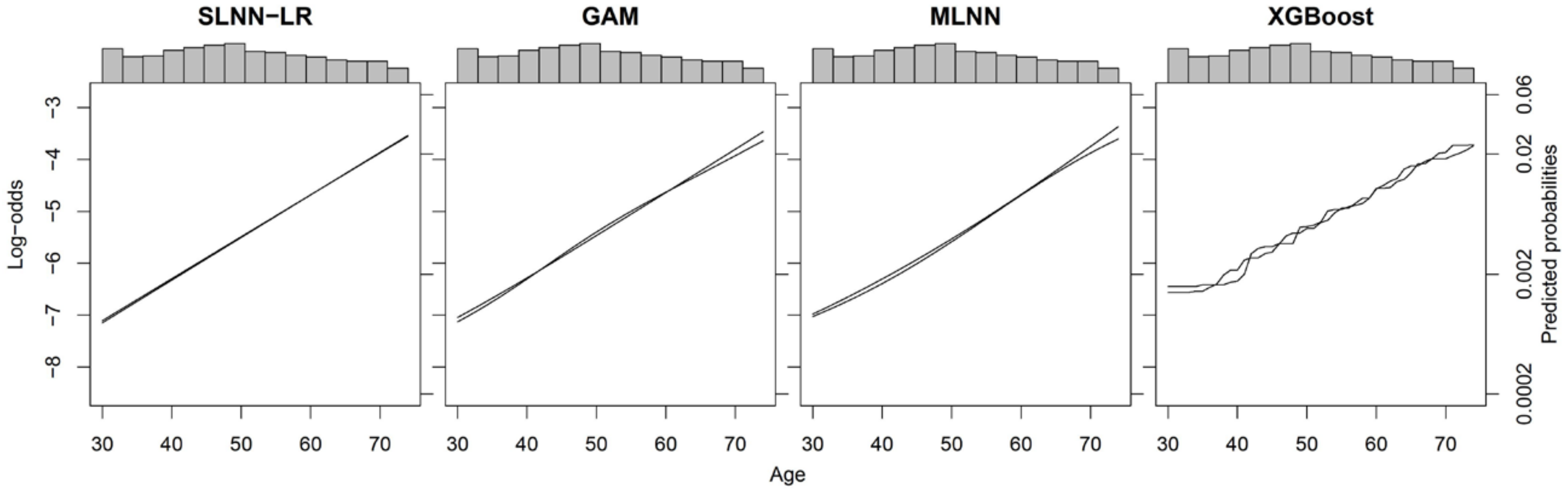
SLNN−LR
GAM
MLNN
XGBoost
Log−odds
−3
−4
−5
−6
−7
−8
30 40 50 60 70
Age
Predicted probabilities
0.06
0.02
0.002
0.0002

C)

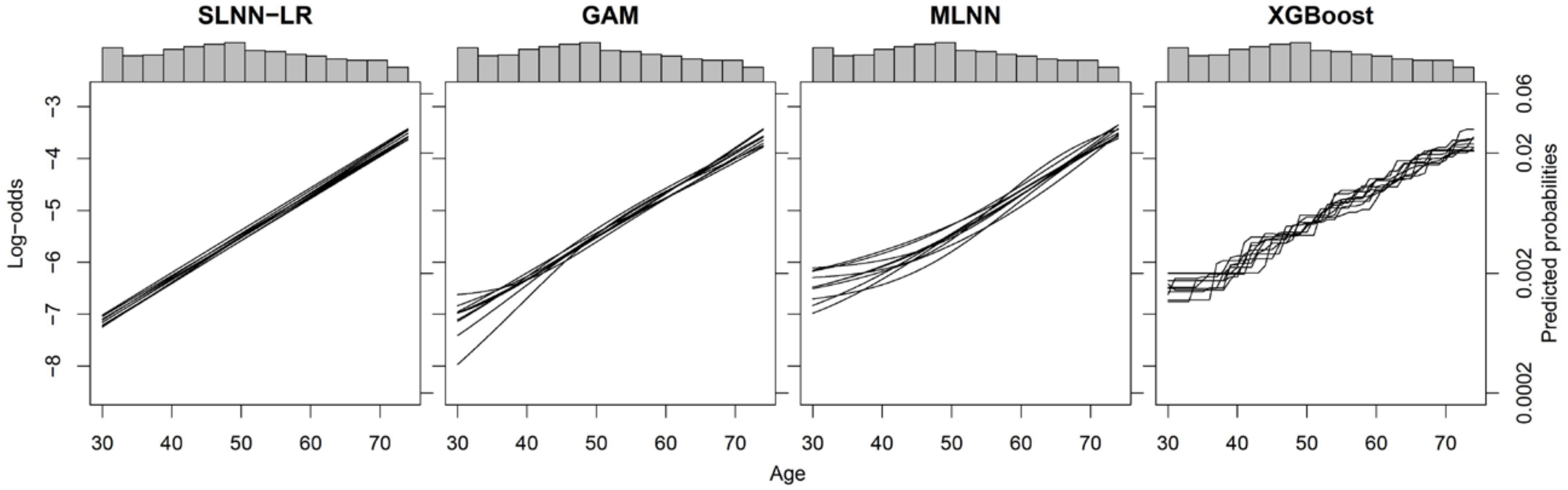
SLNN−LR
GAM
MLNN
XGBoost
Log−odds
−3
−4
−5
−6
−7
−8
30 40 50 60 70
Age
Predicted probabilities
0.06
0.02
0.002
0.0002

D)

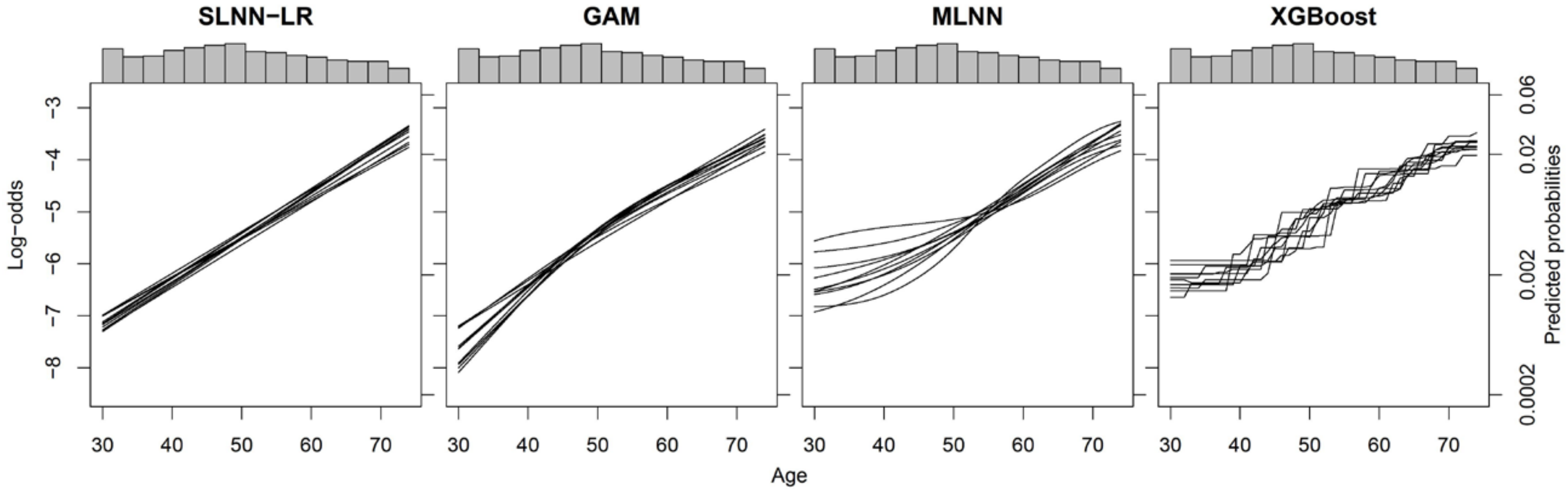
SLNN−LR
GAM
MLNN
XGBoost
Log−odds
-3
-4
-5
-6
-7
-8
Predicted probabilities
0.06
0.02
0.002
0.0002
30 40 50 60 70
Age

E)

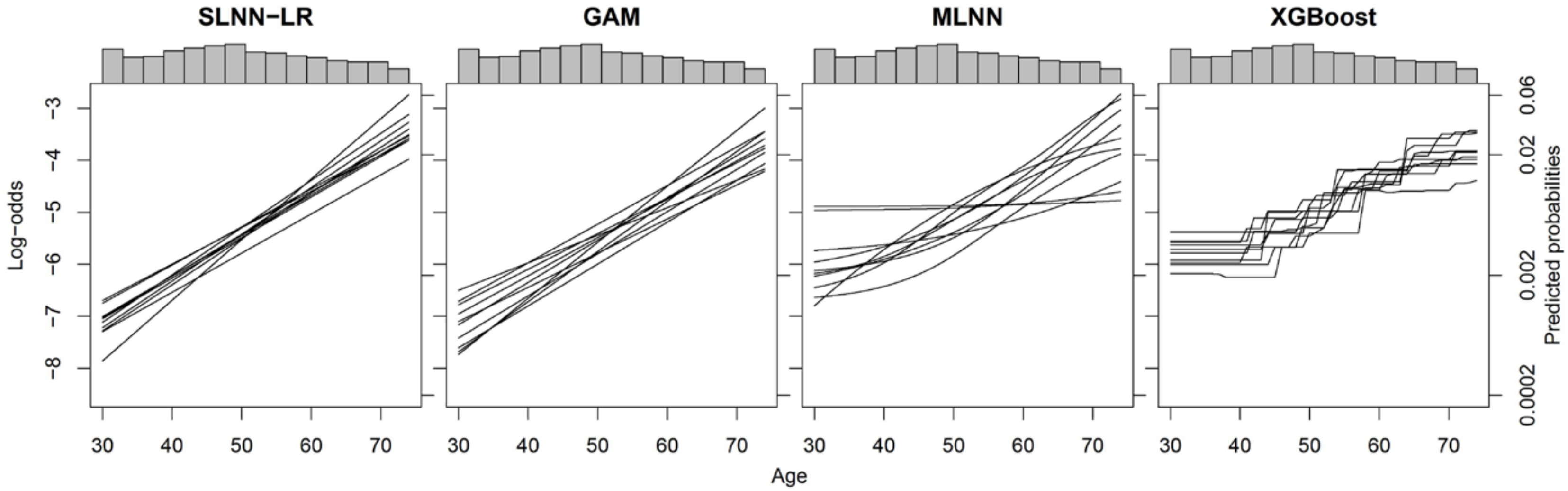
SLNN−LR
GAM
MLNN
XGBoost
Log−odds
-3
-4
-5
-6
-7
-8
Predicted probabilities
0.06
0.02
0.002
0.0002
30 40 50 60 70
Age

Appendix 11: Estimated predictor-risk relation by partial dependence plots for total cholesterol

Partial dependence plots for Total cholesterol in 40-, 50-, 60- and 70-year-old women (red, yellow, green, blue) with all modeling paradigms (SLNN-LR, GAM, MLNN, XGBoost) fitted at A) full data availability, B) data availability of N/2, C) data availability of N/10, D) data availability of N/25 and E) data availability of N/100. In D) and E) ten out of 25 or 100 random models were selected.

Abbreviations: GAM, generalized additive models; MLNN, multi-layer neural networks; SLNN-LR, single-layer neural network/logistic regression; XGBoost, extreme gradient boosted trees;

A)

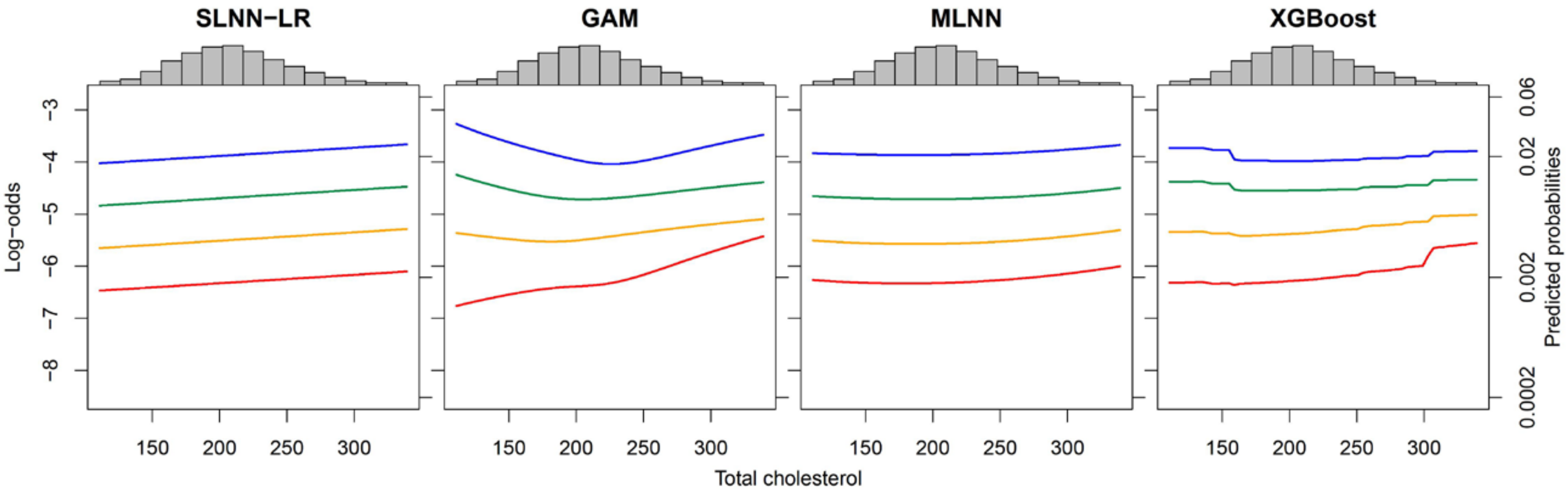

B)

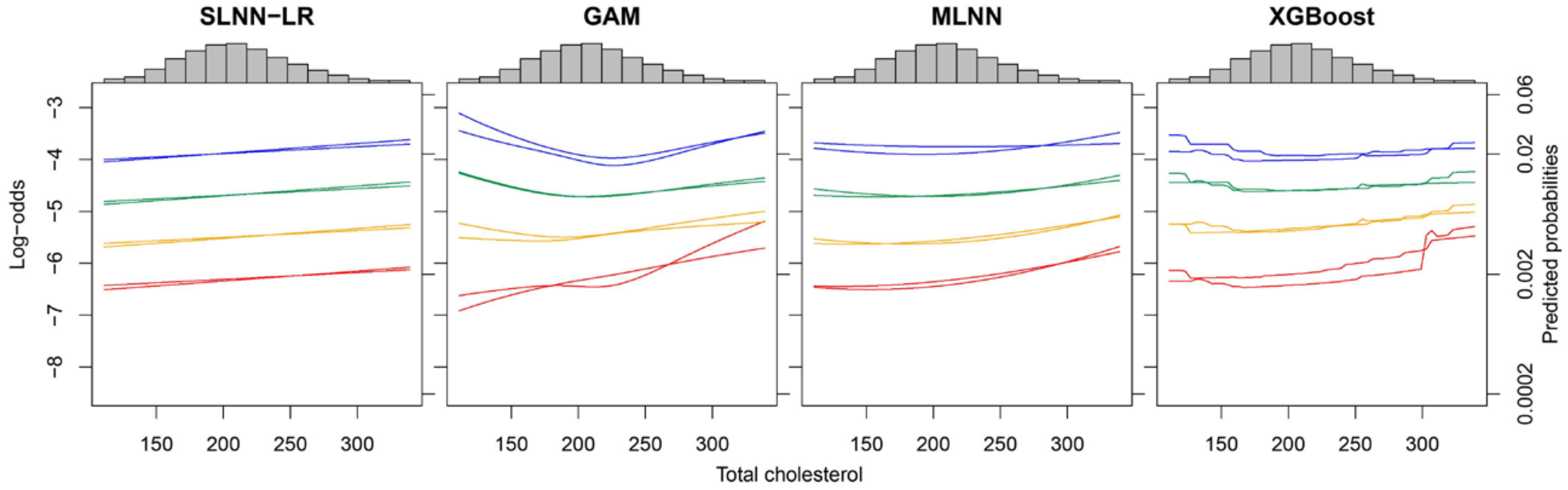

SLNN−LR
GAM
MLNN
XGBoost
Log−odds
−3
−4
−5
−6
−7
−8
150
200
250
300
Total cholesterol
Predicted probabilities
0.06
0.02
0.002
0.0002

C)

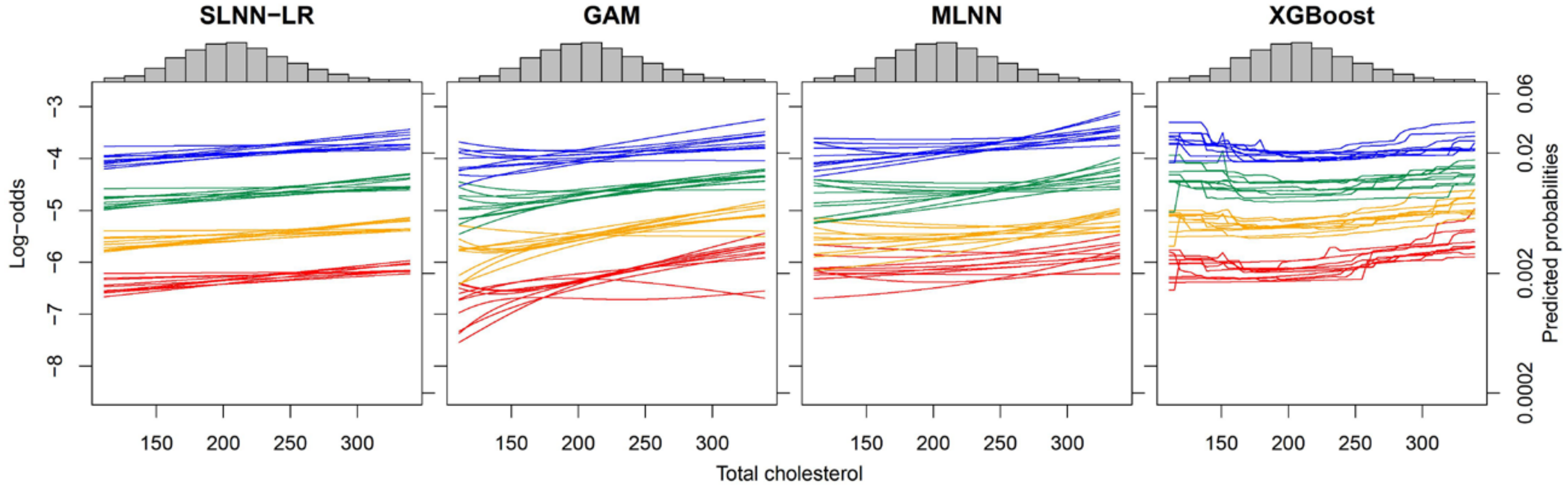

SLNN−LR
GAM
MLNN
XGBoost
Log−odds
−3
−4
−5
−6
−7
−8
150
200
250
300
Total cholesterol
Predicted probabilities
0.06
0.02
0.002
0.0002

D)

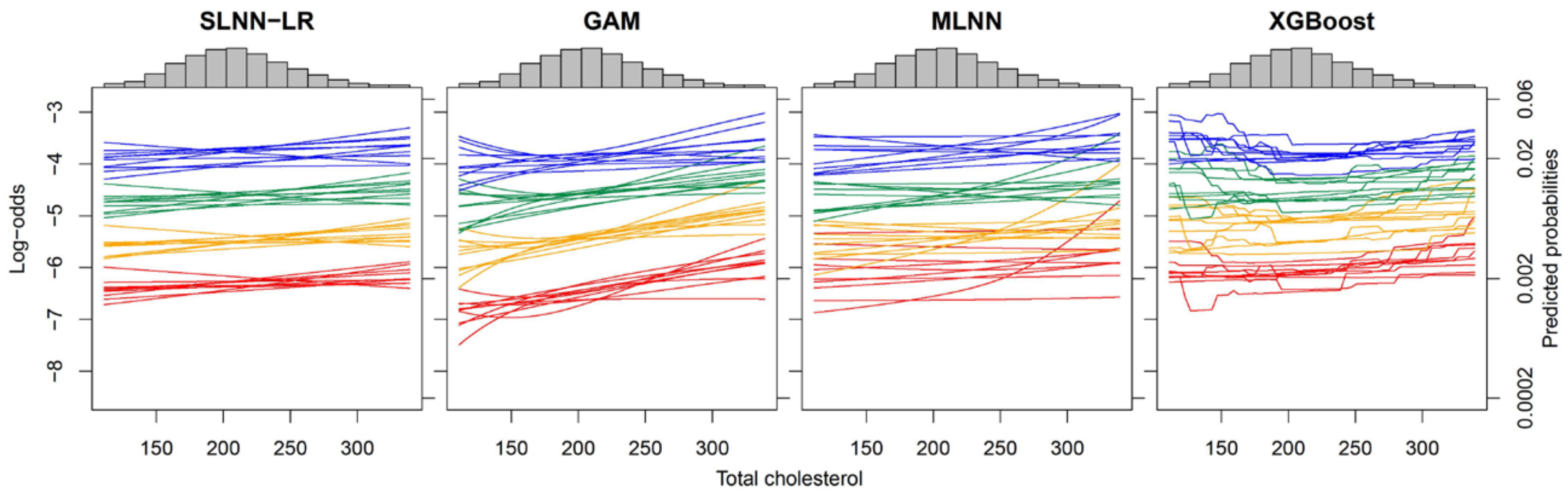
SLNN−LR
GAM
MLNN
XGBoost
Log−odds
−3
−4
−5
−6
−7
−8
150
200
250
300
Total cholesterol
Predicted probabilities
0.06
0.02
0.002
0.0002

E)

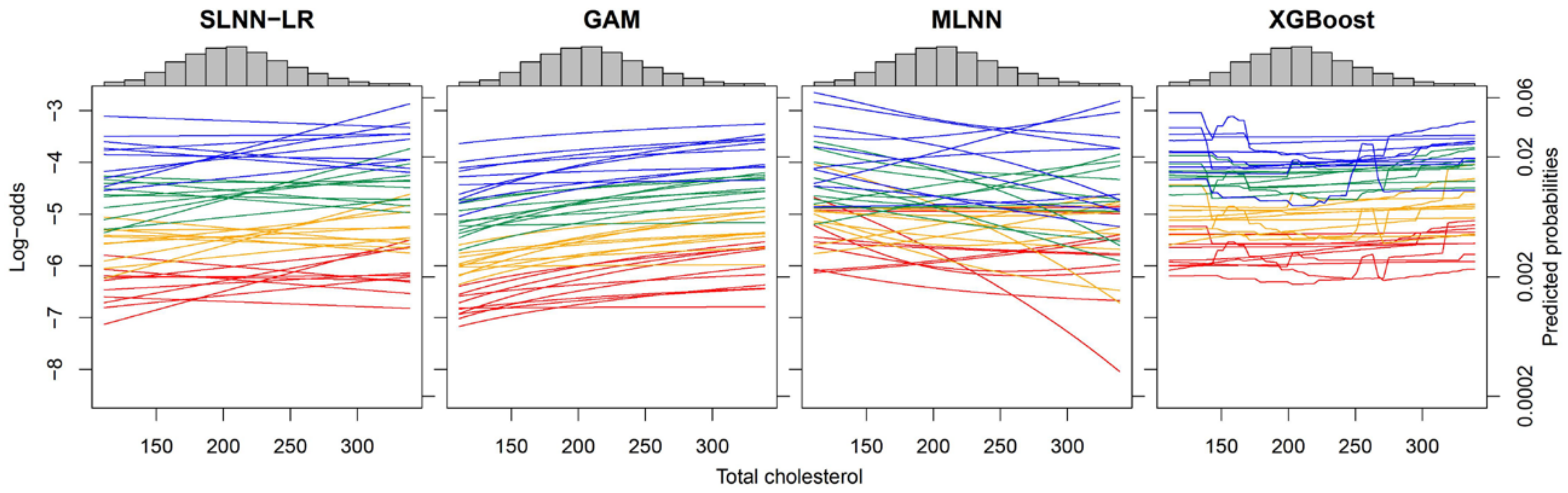
SLNN−LR
GAM
MLNN
XGBoost
Log−odds
−3
−4
−5
−6
−7
−8
150
200
250
300
Total cholesterol
Predicted probabilities
0.06
0.02
0.002
0.0002

Appendix 12: Estimated predictor-risk relation by partial dependence plots for blood glucose

Partial dependence plots for Blood glucose in 40-, 50-, 60- and 70-year-old women (red, yellow, green, blue) with all modeling paradigms (SLNN-LR, GAM, MLNN, XGBoost) fitted at A) full data availability, B) data availability of N/2, C) data availability of N/10, D) data availability of N/25 and E) data availability of N/100. In D) and E) ten out of 25 or 100 random models were selected.

Abbreviations: GAM, generalized additive models; MLNN, multi-layer neural networks; SLNN-LR, single-layer neural network/logistic regression; XGBoost, extreme gradient boosted trees;

A)

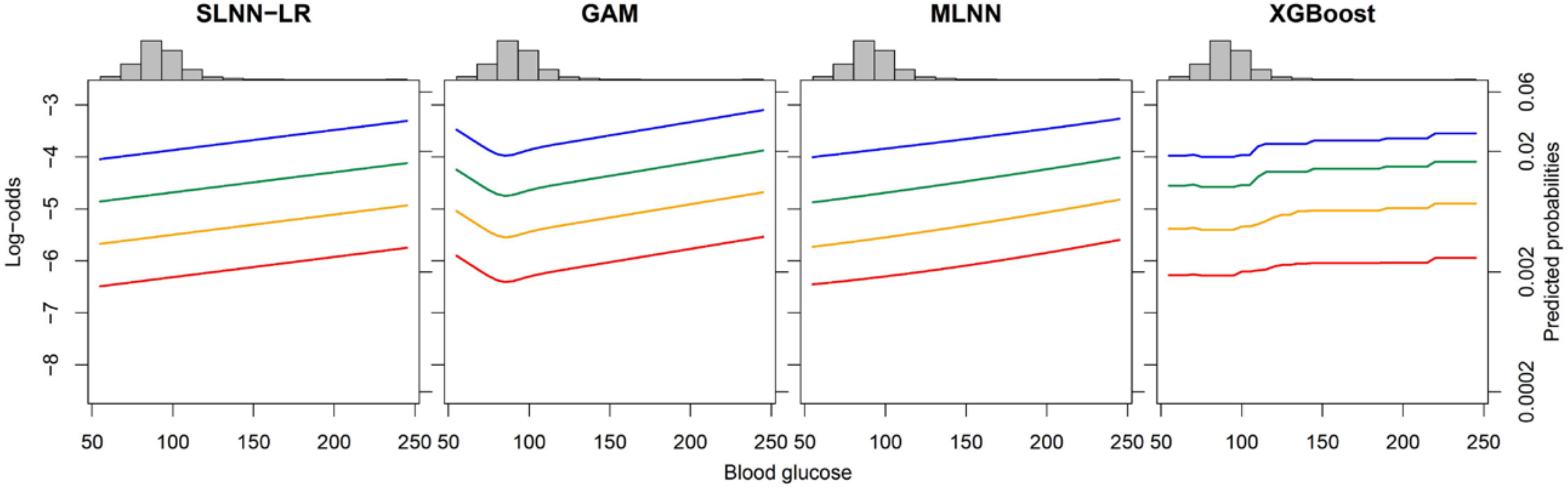

B)

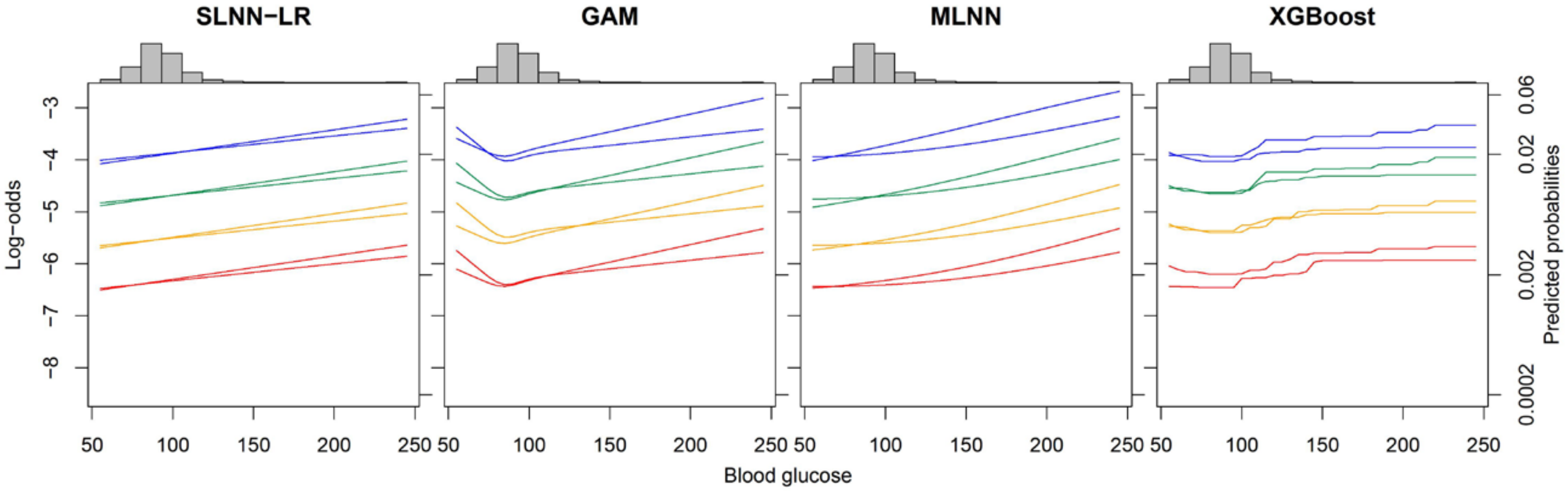
SLNN−LR
GAM
MLNN
XGBoost
Log−odds
-3
-4
-5
-6
-7
-8
50
100
150
200
250
Blood glucose
Predicted probabilities
0.06
0.02
0.002
0.0002

C)

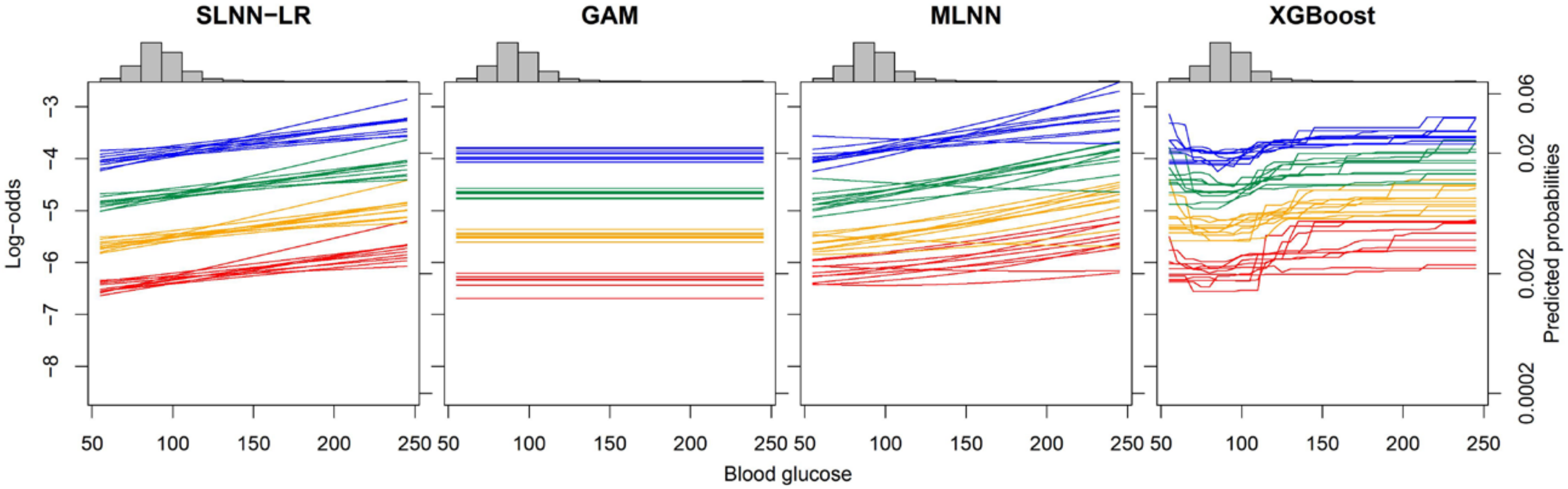
SLNN−LR
GAM
MLNN
XGBoost
Log−odds
-3
-4
-5
-6
-7
-8
50
100
150
200
250
Blood glucose
Predicted probabilities
0.06
0.02
0.002
0.0002

D)

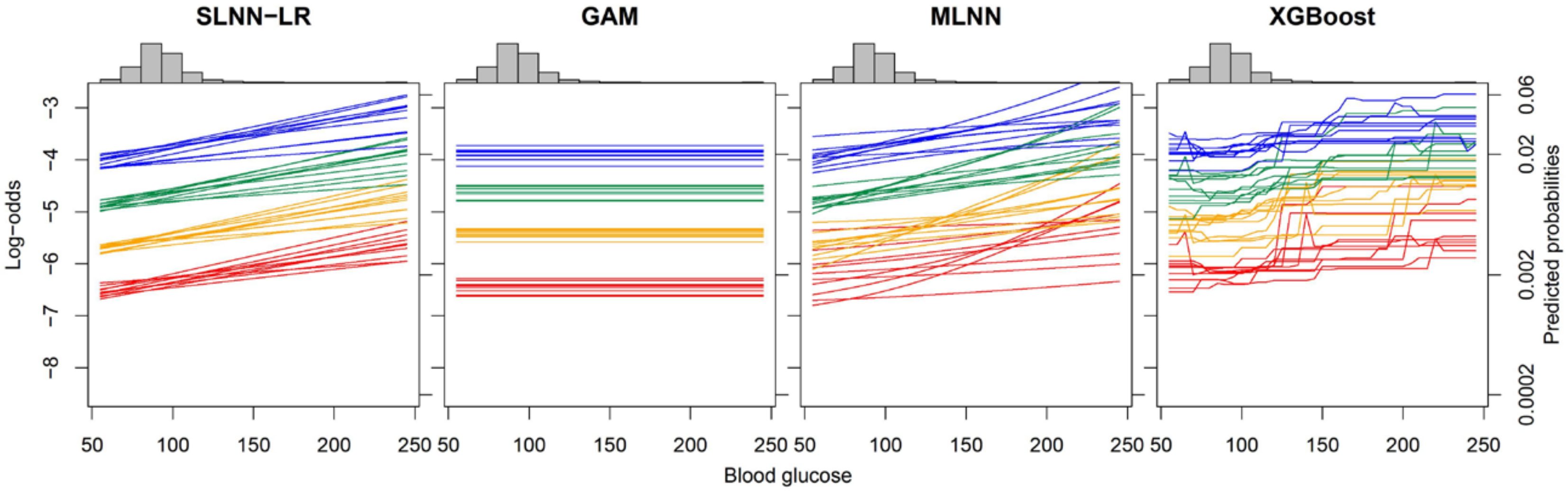
SLNN−LR
GAM
MLNN
XGBoost
Log−odds
-3
-4
-5
-6
-7
-8
0.06
0.02
0.002
0.0002
Predicted probabilities
50 100 150 200 250
Blood glucose

E)

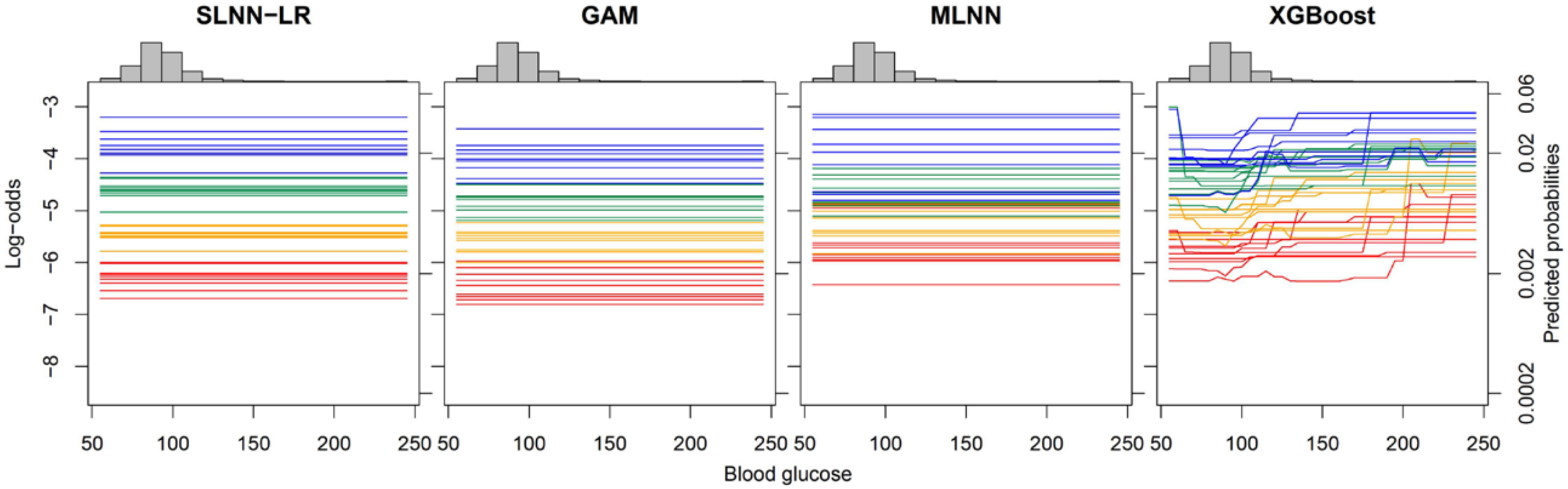
SLNN−LR
GAM
MLNN
XGBoost
Log−odds
-3
-4
-5
-6
-7
-8
0.06
0.02
0.002
0.0002
Predicted probabilities
50 100 150 200 250
Blood glucose

Appendix 13: Estimated predictor-risk relation by partial dependence plots for body mass index

Partial dependence plots for Body mass index (BMI) in 40-, 50-, 60- and 70-year-old women (red, yellow, green, blue) with all modeling paradigms (SLNN-LR, GAM, MLNN, XGBoost) fitted at A) full data availability, B) data availability of N/2, C) data availability of N/10, D) data availability of N/25 and E) data availability of N/100. In D) and E) ten out of 25 or 100 random models were selected.

Abbreviations: GAM, generalized additive models; MLNN, multi-layer neural networks; SLNN-LR, single-layer neural network/logistic regression; XGBoost, extreme gradient boosted trees;

A)

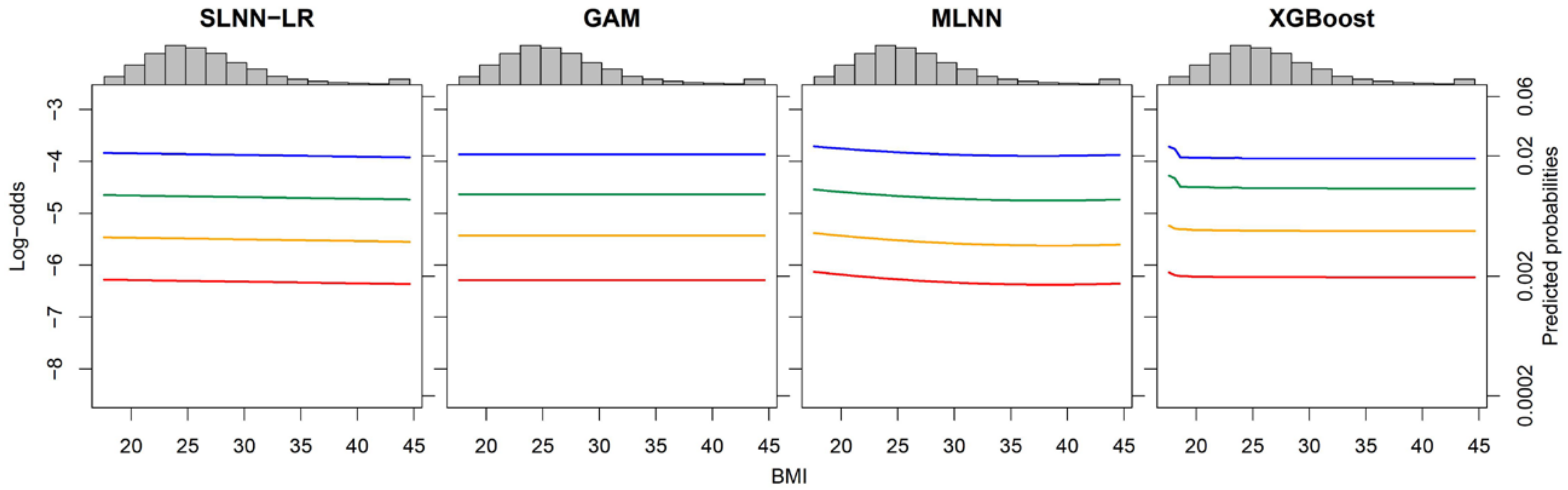

B)

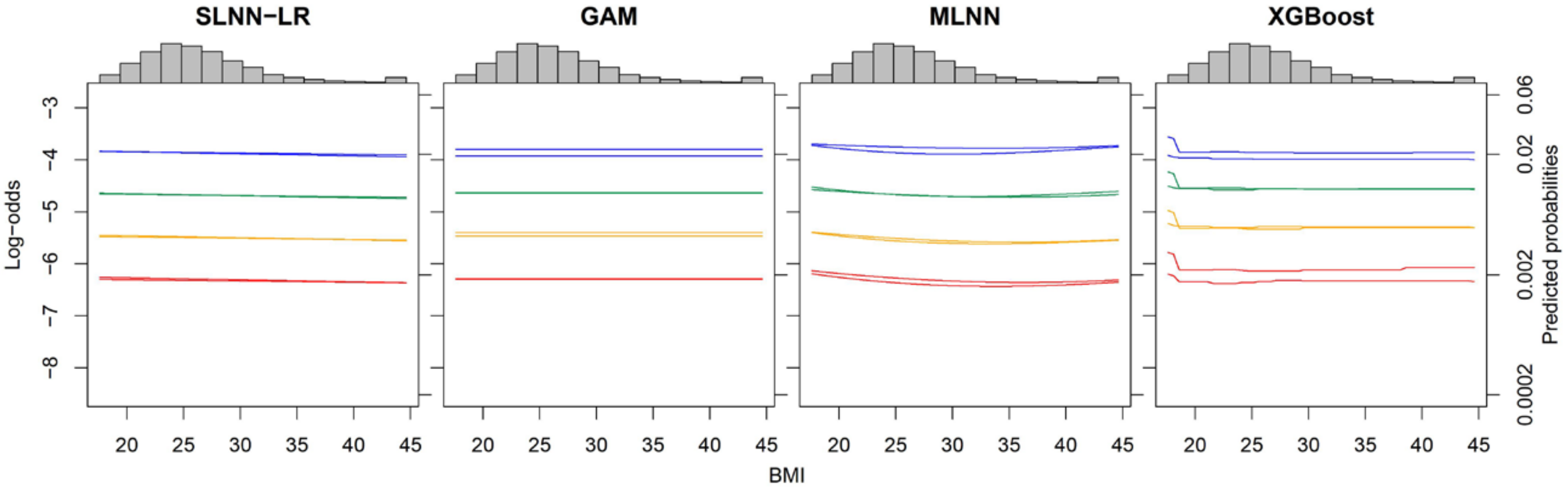
SLNN−LR
GAM
MLNN
XGBoost
Log−odds
−3
−4
−5
−6
−7
−8
20 25 30 35 40 45
BMI
Predicted probabilities
0.06
0.02
0.002
0.0002

C)

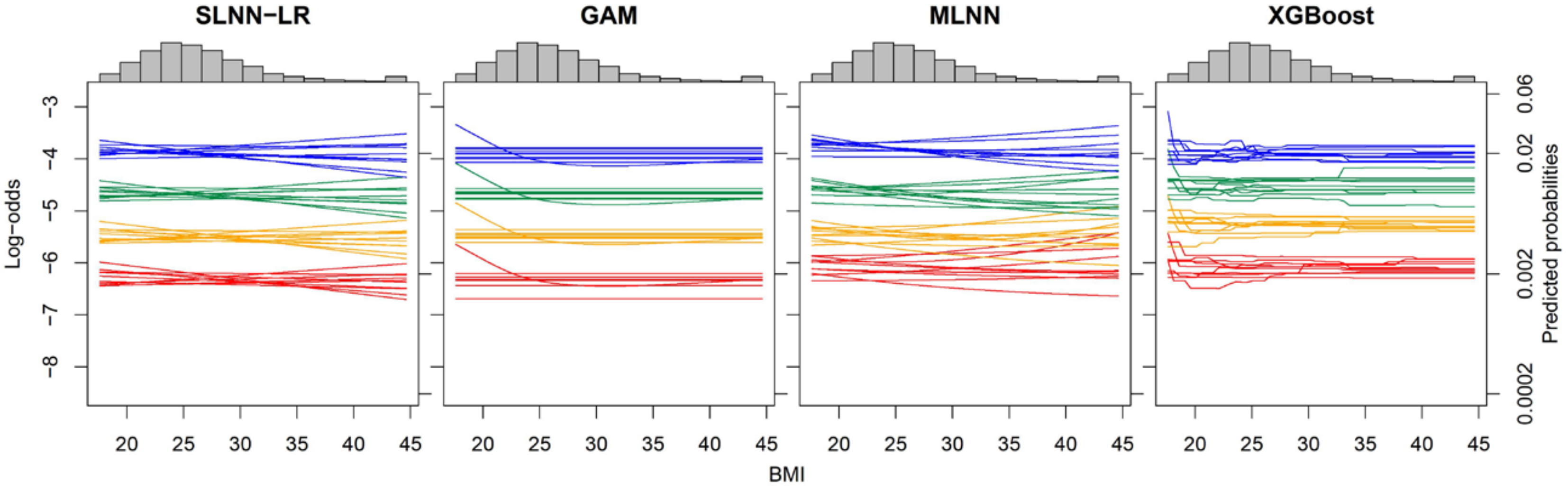
SLNN−LR
GAM
MLNN
XGBoost
Log−odds
−3
−4
−5
−6
−7
−8
20 25 30 35 40 45
BMI
Predicted probabilities
0.06
0.02
0.002
0.0002

D)

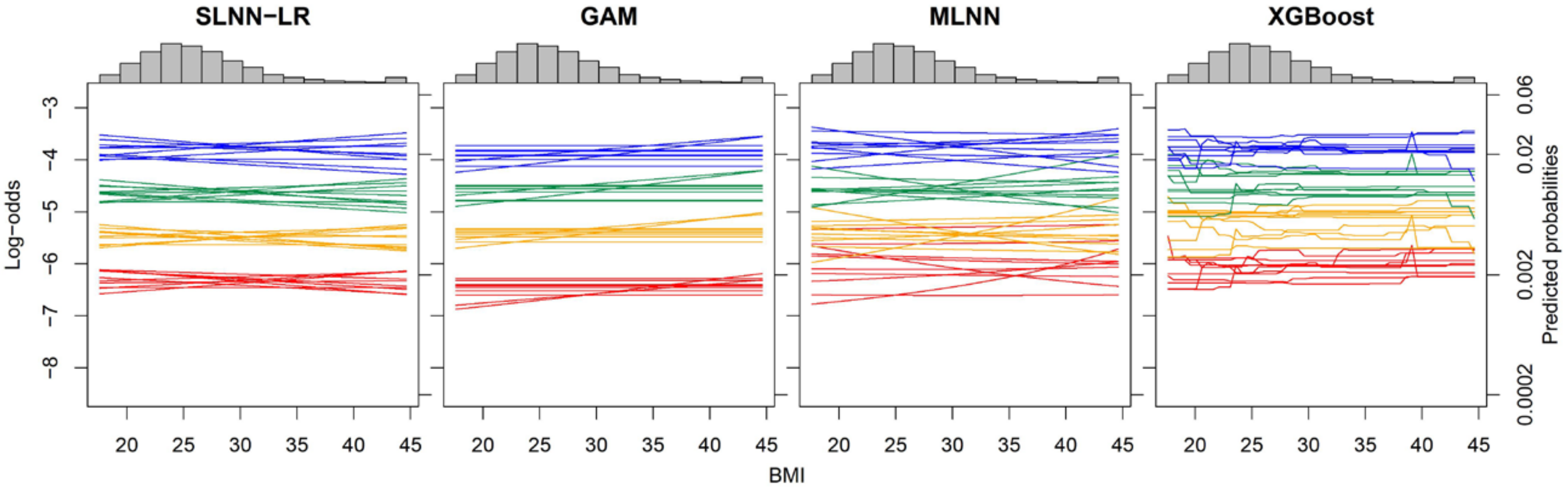

SLNN−LR
GAM
MLNN
XGBoost
Log−odds
Predicted probabilities
BMI

E)

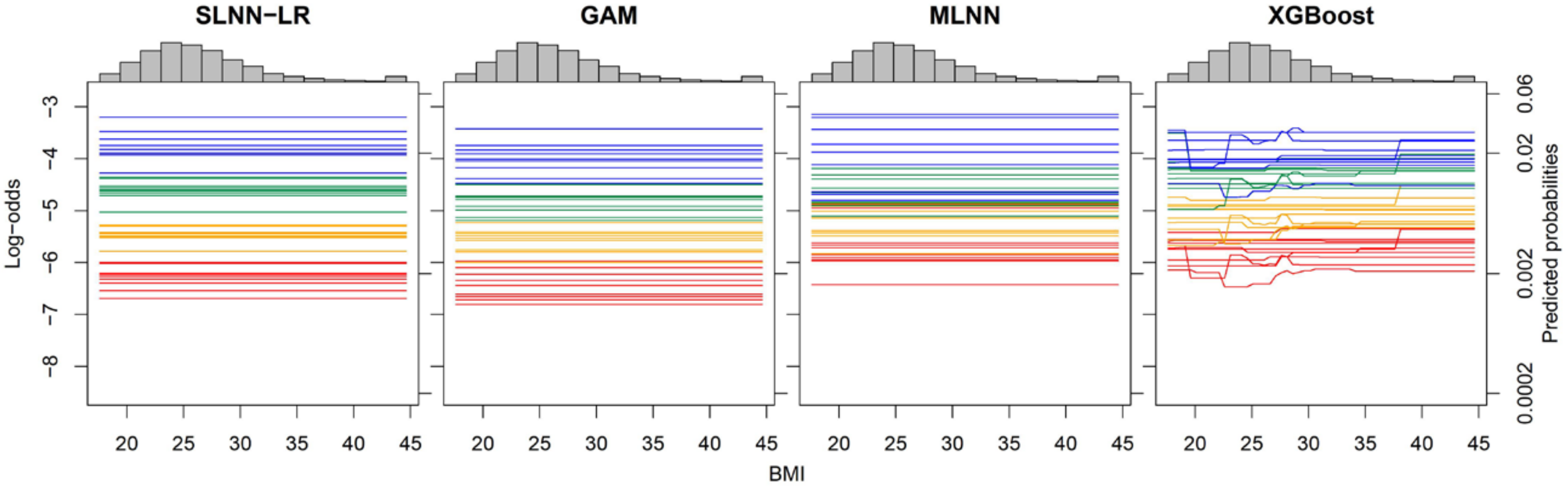

SLNN−LR
GAM
MLNN
XGBoost
Log−odds
Predicted probabilities
BMI
