# Supplementary material for: The roles of predictors in cardiovascular risk models - a question of modeling culture?
Source: BMC Med Res Methodol. 2021 Dec 18;21:284. doi: 10.1186/s12874-021-01487-4 (PMC8684157; doi:10.1186/s12874-021-01487-4)
Supplement: Supplementary file 3 — Additional file 3. Risk prediction of a CVD event within 1 year. [file 12874_2021_1487_MOESM3_ESM.pdf]

The roles of predictors in cardiovascular risk models - a question of modeling culture?

Authors

Christine Wallisch (1), Asan Agibetov (2), Daniela Dunkler (1), Maria Haller (1,3), Matthias Samwald (2), Georg Dorffner (2), Georg Heinze (1)

Affiliations

(1) Section for Clinical Biometrics, Center for Medical Statistics, Informatics and Intelligent Systems, Medical University of Vienna, Austria
(2) Section for Artificial Intelligence and Decision Support, Center for Medical Statistics, Informatics and Intelligent Systems, Medical University of Vienna, Austria
(3) Ordensklinikum Linz, Hospital Elisabethinen, Department of Nephrology, Linz, Austria

Additional file 3: Risk prediction of a CVD event within 1 year

In the following we present the risk equations estimated with generalized additive models on the full training data set to prognosticate the risk of a CVD event within 1 year from baseline. The definition of the predictors is explained in the main manuscript. Two separate risk equations were estimated for men and for women.

The risk equations estimate the risk of a CVD event (Prob{CVD=1}) within 1 year from baseline.

Risk equation for men

$$\text{Prob}\{\text{CVD} = 1\} = \frac{1}{1 + \exp(-X\beta)}, \quad \text{where}$$

$$
\begin{aligned}
X\hat{\beta} = \\
& 6.927891 \\
& +0.04463686\text{age} - 1.904999\times10^{-5}(\text{age} - 34)_+^3 + 3.492498\times10^{-5}(\text{age} - 49)_+^3 \\
& -1.587499\times10^{-5}(\text{age} - 67)_+^3 \\
& -0.4054836\ln(\text{total.cholesterol}) + 29.53088(\ln(\text{total.cholesterol}) - 4.976734)_+^3 \\
& -121.2538(\ln(\text{total.cholesterol}) - 5.257495)_+^3 \\
& +116.7356(\ln(\text{total.cholesterol}) - 5.411646)_+^3 \\
& -25.01277(\ln(\text{total.cholesterol}) - 5.645447)_+^3 \\
& -0.04666346\text{bmiscore} + 0.0007101575(\text{bmiscore} - 21.5)_+^3 \\
& -0.00177571(\text{bmiscore} - 25.2)_+^3 + 0.001124099(\text{bmiscore} - 28.1)_+^3 \\
& -5.854676\times10^{-5}(\text{bmiscore} - 36)_+^3 \\
& -2.164861\ln(\text{hdl.cholesterol}) - 2.417759(\ln(\text{hdl.cholesterol}) - 3.433987)_+^3 \\
& +26.93652(\ln(\text{hdl.cholesterol}) - 3.806662)_+^3 \\
& -35.6179(\ln(\text{hdl.cholesterol}) - 4.007333)_+^3 \\
& +11.09913(\ln(\text{hdl.cholesterol}) - 4.369448)_+^3 \\
& +0.06554149[\text{Waist.size.too.large}] \\
& -0.3036254[\text{Smoking.no}] \\
& -0.1173022[\text{Physical.activity.sometimes}] \\
& -0.2117443[\text{Physical.activity.regularly}] \\
& -0.02531492\text{systolic.bloodpressure} \\
& +2.996185\times10^{-5}(\text{systolic.bloodpressure} - 110)_+^3 \\
& -7.15403\times10^{-5}(\text{systolic.bloodpressure} - 125)_+^3 \\
& +4.495223\times10^{-5}(\text{systolic.bloodpressure} - 138)_+^3 \\
& -3.373779\times10^{-6}(\text{systolic.bloodpressure} - 165)_+^3 \\
& -3.650783[\text{Diabetes.no}] \\
& -0.0157408\text{diastolic.bloodpressure} \\
& +2.012677\times10^{-5}(\text{diastolic.bloodpressure} - 70)_+^3 \\
& -1.384448\times10^{-5}(\text{diastolic.bloodpressure} - 80)_+^3 \\
& -2.179423\times10^{-5}(\text{diastolic.bloodpressure} - 85)_+^3 \\
& +1.551194\times10^{-5}(\text{diastolic.bloodpressure} - 100)_+^3 \\
& -3.87149[\text{Bloodpressure.medication.no}] \\
& -0.03253383\text{blood.glucose} + 2.447313\times10^{-5}(\text{blood.glucose} - 72)_+^3 \\
& -6.231339\times10^{-5}(\text{blood.glucose} - 89)_+^3 + 3.784565\times10^{-5}(\text{blood.glucose} - 100)_+^3 \\
& -5.392406\times10^{-9}(\text{blood.glucose} - 137)_+^3 \\
& +0.003422428\text{triglycerides} - 2.719901\times10^{-7}(\text{triglycerides} - 55)_+^3 \\
& +4.749407\times10^{-7}(\text{triglycerides} - 97)_+^3 - 1.981957\times10^{-7}(\text{triglycerides} - 149)_+^3
\end{aligned}
$$

$$-4.754933\times10^{-9}(\text{triglycerides}-332)_+^3$$
$$-0.1121896[\text{Urine.positive}]$$
$$+0.2399674[\text{Protein.positive}]$$
$$+\text{age}[-0.0017875\ln(\text{total.cholesterol})$$
$$-0.5934824(\ln(\text{total.cholesterol})-4.976734)_+^3$$
$$+2.740139(\ln(\text{total.cholesterol})-5.257495)_+^3$$
$$-2.849313(\ln(\text{total.cholesterol})-5.411646)_+^3$$
$$+0.7026559(\ln(\text{total.cholesterol})-5.645447)_+^3]$$
$$+\text{age}'[-0.00623635\ln(\text{total.cholesterol})$$
$$+0.533249(\ln(\text{total.cholesterol})-4.976734)_+^3$$
$$-2.709571(\ln(\text{total.cholesterol})-5.257495)_+^3$$
$$+2.970869(\ln(\text{total.cholesterol})-5.411646)_+^3$$
$$-0.7945467(\ln(\text{total.cholesterol})-5.645447)_+^3]$$
$$+\text{age}[0.03497072\ln(\text{hdl.cholesterol})$$
$$+0.01118576(\ln(\text{hdl.cholesterol})-3.433987)_+^3$$
$$-0.3317763(\ln(\text{hdl.cholesterol})-3.806662)_+^3$$
$$+0.4867381(\ln(\text{hdl.cholesterol})-4.007333)_+^3$$
$$-0.1661476(\ln(\text{hdl.cholesterol})-4.369448)_+^3]$$
$$+\text{age}'[-0.01502551\ln(\text{hdl.cholesterol})$$
$$+0.02857156(\ln(\text{hdl.cholesterol})-3.433987)_+^3$$
$$+0.05253031(\ln(\text{hdl.cholesterol})-3.806662)_+^3$$
$$-0.1554504(\ln(\text{hdl.cholesterol})-4.007333)_+^3$$
$$+0.0743485(\ln(\text{hdl.cholesterol})-4.369448)_+^3]$$
$$+[\text{Smoking.no}][-0.009093511\text{age}+1.721865\times10^{-5}(\text{age}-34)_+^3$$
$$-3.156752\times10^{-5}(\text{age}-49)_+^3+1.434887\times10^{-5}(\text{age}-67)_+^3]$$
$$+[\text{Diabetes.no}][0.01477648\text{age}+5.117527\times10^{-6}(\text{age}-34)_+^3$$
$$-9.382132\times10^{-6}(\text{age}-49)_+^3+4.264606\times10^{-6}(\text{age}-67)_+^3]$$
$$+[\text{Bloodpressure.medication.no}][0.004192558\text{diastolic.bloodpressure}$$
$$+1.247454\times10^{-5}(\text{diastolic.bloodpressure}-70)_+^3$$
$$-8.471895\times10^{-5}(\text{diastolic.bloodpressure}-80)_+^3$$
$$+8.800952\times10^{-5}(\text{diastolic.bloodpressure}-85)_+^3$$
$$-1.576511\times10^{-5}(\text{diastolic.bloodpressure}-100)_+^3]$$
$$+[\text{Bloodpressure.medication.no}][0.02602349\text{systolic.bloodpressure}$$
$$-2.628245\times10^{-5}(\text{systolic.bloodpressure}-110)_+^3$$
$$+6.80811\times10^{-5}(\text{systolic.bloodpressure}-125)_+^3$$
$$-4.732256\times10^{-5}(\text{systolic.bloodpressure}-138)_+^3$$
$$+5.523913\times10^{-6}(\text{systolic.bloodpressure}-165)_+^3]$$
$$+[\text{Diabetes.no}][0.02464715\text{blood.glucose}$$
$$-1.012653\times10^{-5}(\text{blood.glucose}-72)_+^3+2.023176\times10^{-5}(\text{blood.glucose}-89)_+^3$$
$$-8.456762\times10^{-6}(\text{blood.glucose}-100)_+^3-1.64847\times10^{-6}(\text{blood.glucose}-137)_+^3]$$

and $[c]=1$ if subject is in group c, 0 otherwise; $(x)_+=x$ if $x>0$, 0 otherwise\

Risk equation for women

$$\text{Prob}\{\text{CVD} = 1\} = \frac{1}{1 + \exp(-X\beta)}, \quad \text{where}$$

$$
\begin{aligned}
X\hat{\beta} = &\\
& -33.65301 \\
& +0.7322975\text{age} - 0.0003147659(\text{age} - 33)_+^3 + 0.000579832(\text{age} - 49)_+^3 \\
& -0.000265066(\text{age} - 68)_+^3 \\
& +6.547769\ln(\text{total.cholesterol}) - 36.80792(\ln(\text{total.cholesterol}) - 5.003946)_+^3 \\
& +209.481(\ln(\text{total.cholesterol}) - 5.267858)_+^3 \\
& -238.8839(\ln(\text{total.cholesterol}) - 5.4161)_+^3 \\
& +66.2108(\ln(\text{total.cholesterol}) - 5.655992)_+^3 \\
& -0.03297816[\text{Physical.activity.sometimes}] \\
& -0.1928575[\text{Physical.activity.regularly}] \\
& -2.04902\ln(\text{hdl.cholesterol}) + 13.50757(\ln(\text{hdl.cholesterol}) - 3.663562)_+^3 \\
& -61.95116(\ln(\text{hdl.cholesterol}) - 4.025352)_+^3 \\
& +64.26815(\ln(\text{hdl.cholesterol}) - 4.234107)_+^3 \\
& -15.82456(\ln(\text{hdl.cholesterol}) - 4.564348)_+^3 \\
& -0.01935856\text{blood.glucose} + 8.25009\times10^{-6}(\text{blood.glucose} - 70)_+^3 \\
& -8.439467\times10^{-6}(\text{blood.glucose} - 85)_+^3 - 4.323867\times10^{-6}(\text{blood.glucose} - 95)_+^3 \\
& +4.513244\times10^{-6}(\text{blood.glucose} - 122)_+^3 \\
& -0.9281799[\text{Smoking.no}] \\
& -0.7353138[\text{Diabetes.no}] \\
& +0.004199504\text{systolic.bloodpressure} \\
& -6.363074\times10^{-6}(\text{systolic.bloodpressure} - 100)_+^3 \\
& +2.906471\times10^{-5}(\text{systolic.bloodpressure} - 120)_+^3 \\
& -2.591954\times10^{-5}(\text{systolic.bloodpressure} - 130)_+^3 \\
& +3.217901\times10^{-6}(\text{systolic.bloodpressure} - 161)_+^3 \\
& +0.2980217[\text{Protein.positive}] \\
& -2.208068[\text{Bloodpressure.medication.no}] \\
& +\text{age}[-0.1461906\ln(\text{total.cholesterol}) \\
& +0.8759523(\ln(\text{total.cholesterol}) - 5.003946)_+^3 \\
& -4.780949(\ln(\text{total.cholesterol}) - 5.267858)_+^3 \\
& +5.354449(\ln(\text{total.cholesterol}) - 5.4161)_+^3 \\
& -1.449453(\ln(\text{total.cholesterol}) - 5.655992)_+^3] \\
& +\text{age}'[0.09110886\ln(\text{total.cholesterol}) \\
& -0.8907257(\ln(\text{total.cholesterol}) - 5.003946)_+^3 \\
& +5.168119(\ln(\text{total.cholesterol}) - 5.267858)_+^3 \\
& -5.940718(\ln(\text{total.cholesterol}) - 5.4161)_+^3 \\
& +1.663325(\ln(\text{total.cholesterol}) - 5.655992)_+^3] \\
& +\text{age}[0.03295924\ln(\text{hdl.cholesterol}) \\
& -0.3237263(\ln(\text{hdl.cholesterol}) - 3.663562)_+^3 \\
& +1.447591(\ln(\text{hdl.cholesterol}) - 4.025352)_+^3
\end{aligned}
$$

$$
\begin{aligned}
&-1.479638(\ln(\text{hdl.cholesterol}) - 4.234107)_+^3 \\
&+0.3557733(\ln(\text{hdl.cholesterol}) - 4.564348)_+^3] \\
&+\text{age}'[-0.03133409\ln(\text{hdl.cholesterol}) \\
&+0.3365619(\ln(\text{hdl.cholesterol}) - 3.663562)_+^3 \\
&-1.408993(\ln(\text{hdl.cholesterol}) - 4.025352)_+^3 \\
&+1.38163(\ln(\text{hdl.cholesterol}) - 4.234107)_+^3 \\
&-0.309199(\ln(\text{hdl.cholesterol}) - 4.564348)_+^3] \\
&+[\text{Smoking.no}][0.003656776\text{age} + 4.555613\times 10^{-6}(\text{age} - 33)_+^3 \\
&-8.391918\times 10^{-6}(\text{age} - 49)_+^3 + 3.836305\times 10^{-6}(\text{age} - 68)_+^3] \\
&+[\text{Diabetes.no}][-0.0006506475\text{blood.glucose} \\
&+2.161183\times 10^{-5}(\text{blood.glucose} - 70)_+^3 - 7.36905\times 10^{-5}(\text{blood.glucose} - 85)_+^3 \\
&+5.93605\times 10^{-5}(\text{blood.glucose} - 95)_+^3 - 7.281826\times 10^{-6}(\text{blood.glucose} - 122)_+^3] \\
&+[\text{Diabetes.no}][-0.001802767\text{age} + 1.228361\times 10^{-5}(\text{age} - 33)_+^3 \\
&-2.262771\times 10^{-5}(\text{age} - 49)_+^3 + 1.034409\times 10^{-5}(\text{age} - 68)_+^3] \\
&+[\text{Bloodpressure.medication.no}][0.01364413\text{systolic.bloodpressure} \\
&+1.089441\times 10^{-7}(\text{systolic.bloodpressure} - 100)_+^3 \\
&-8.987619\times 10^{-6}(\text{systolic.bloodpressure} - 120)_+^3 \\
&+1.167248\times 10^{-5}(\text{systolic.bloodpressure} - 130)_+^3 \\
&-2.793802\times 10^{-6}(\text{systolic.bloodpressure} - 161)_+^3]
\end{aligned}
$$

and $[c] = 1$ if subject is in group c, 0 otherwise; $(x)_+ = x$ if $x > 0$, 0 otherwise\
